# Co-targeting ferroptosis and immune evasion through small molecules in breast cancer

**DOI:** 10.1515/jtim-2025-0093

**Published:** 2025-12-22

**Authors:** Jianbo Zhou, Wangji Yang, Hailin Tang, Yutian Zou, Zhaokai Zhou, Cheng Peng, Fu Peng

**Affiliations:** West China School of Pharmacy, West China School of Medicine, Department of Thoracic Surgery and Institute of Thoracic Oncology, Frontiers Science Center for Disease-Related Molecular Network, West China Hospital, Sichuan University, Chengdu, Sichuan Province, China; State Key Laboratory of Southwestern Chinese Medicine Resources, Chengdu University of Traditional Chinese Medicine, Chengdu, Sichuan Province, China; State Key Laboratory of Oncology in South China, Guangdong Provincial Clinical Research Center for Cancer, Sun Yat-Sen University Cancer Center, Guangzhou, Guangdong Province, China; Department of Urology, National Clinical Research Center for Metabolic Diseases, The Second Xiangya Hospital of Central South University, Changsha, Hunan Province, China; Key Laboratory of Drug-Targeting and Drug Delivery System of the Education Ministry, Sichuan Engineering Laboratory for Plant-Sourced Drug and Sichuan Research Center for Drug Precision Industrial Technology, Sichuan University, Chengdu, Sichuan Province, China

**Keywords:** breast cancer, ferroptosis, small molecules, natural products, tumor microenvironment

## Abstract

Breast cancer accounts for the highest proportion of cancer cases among women worldwide. Despite remarkable advances in cancer diagnosis and treatment, novel precision therapy strategies for various subtypes of breast cancer are urgently needed. Ferroptosis, which is different from programmed cell death, such as apoptosis, necrosis, and pyroptosis, is considered an alternative method for cancer therapy. A comprehensive understanding of ferroptosis in breast cancer is lacking, including, but not limited to, ferroptotic inducers (small molecule drugs and natural products) and the interaction between ferroptosis and immunotherapy. Induction of ferroptosis is recognized as a novel and promising strategy for cancer pharmacotherapy, and a comprehensive understanding of the role of ferroptosis in breast cancer could help provide alternative treatment strategies for breast cancer. Furthermore, we highlight the signaling crosstalk bridging ferroptosis with the immune microenvironment and the feasibility of targeting their potential regulators (*e.g*., STAT3, AR, EZH2, and PRMT5) using small molecules to simultaneously achieve the induction of ferroptosis and inhibition of immune escape.

## Introduction

Despite of encouraging advancements in prevention and treatment of breast cancer in recent years, its incidence and mortality remain alarmingly high. Breast cancer is the most prevalent type of among newly diagnosed cases in women, accounting for 11.7% of all cancer diagnoses globally.^[1,2]^ The latest epidemiological statistics of breast cancer imply that nearly one-third of newly diagnosed cancers in women is breast cancer, comprising 14% of cancer-related deaths among them.^[3]^ Recent data indicate that the diagnosis and mortality rate for breast cancer is 30% and 22%, respectively in the United States in 2021.^[4]^ Furthermore, a trend of younger patients being diagnosed with breast cancer deserves concern.^[5]^ Breast cancer can be classified into three histological subtypes based on the expression status of estrogen receptor (ER), progesterone receptor (PR), and human epidermal growth factor receptor 2 (HER2): luminal (positive for ER and/or PR, HER2 negative), HER2+ (HER2 positive), and triple-negative breast cancer (TNBC; negative for HER2, ER, and PR). The luminal subtype can be further divided into luminal A (low Ki-67 expression) and luminal B (high Ki-67 expression) based on Ki-67 levels.^[6,7]^ TNBC is more frequently observed in younger patients and is typically associated with larger tumor size, higher grade, and a greater likelihood of lymph node involvement at diagnosis, demonstrating increased biological aggressiveness.^[8]^ Women with TNBC also experience higher early distant recurrence rates and worse five-year survival outcomes than those with other breast cancer subtypes.^[8,9]^

In 2011, Lehmann *et al*. performed gene expression profiling on tumor samples originating from 587 patients with TNBC with six molecular subtypes identified, say, basal-like 1 (BL1), basal-like 2 (BL2), mesenchymal (M), mesenchymal stem-like (MSL), immunomodulatory (IM), and luminal androgen receptor (LAR).^[10]^ In 2015, Matthew *et al*.^[9]^ analyzed RNA and DNA collected from 198 TNBC tumors at Baylor College of Medicine and distinguished four subtypes: luminal androgen receptor (LAR), mesenchymal (MES), basal-like immunosuppressed (BLIS), and basal-like immune-activated (BLIA).^[11]^ In 2016, an integrative study of TNBC by Shao Zhimin and his colleagues from Fudan University who revealed significant heterogeneity in molecular characteristics, metabolic reprogramming, and tumor microenvironment (TME) proposed a classification system for TNBC that includes four subtypes, known as the Fudan University Shanghai Cancer Center (FUSCC) classification: mesenchymal-like (MES), luminal androgen receptor (LAR), immunomodulatory (IM), basal-like and immune-suppressed (BLIS) subtypes.^[9]^

Although PARP inhibitors have been approved for treating poly (adenosine diphosphate–ribose) polymerase (BRCA)-mutated triple-negative breast cancer (TNBC),^[12]^ targeted therapies for TNBC remain the leading therapies in the early stages, with chemotherapy being the sole standard treatment for non-metastatic TNBC.^[13,14]^ Neoadjuvant chemotherapy (NAC) refers to chemotherapy administered before surgery or radiation therapy, benefiting patients with locally advanced disease through reducing tumor size and increasing the likelihood of breast-conserving surgery.^[15,16]^ NAC shows significantly better efficacy for TNBC subtypes compared to estrogen receptor-positive breast cancer, thereby improving the prognosis of TNBC patients. Preferred chemotherapy regimens for TNBC include combination therapies such as paclitaxel/ docetaxel plus doxorubicin plus cyclophosphamide, docetaxel plus cyclophosphamide, doxorubicin plus cyclophosphamide, and cyclophosphamide plus methotrexate plus fluorouracil.^[17,18]^ While there are currently no universally accepted biomarkers to predict response to NAC, evidence derived from multi-omics or tumor immune microenvironments may serve as potential indicators, including but not limited to proteomic, genomic, and transcriptomic features, as well as non-coding RNAs.^[19,20]^ The introduction of neoadjuvant chemo-immunotherapy has transformed the treatment landscape for TNBC, although challenges such as patient stratification, management of drug toxicity, and identification of biomarkers still remain.^[21]^ Identifying candidate small molecules can provide alternative options for chemotherapy for the treatment of TNBC. Searching for effective and low-toxicity candidate molecules from natural products may be a promising approach.^[22, 23, 24, 25, 26]^

**Figure 1:**
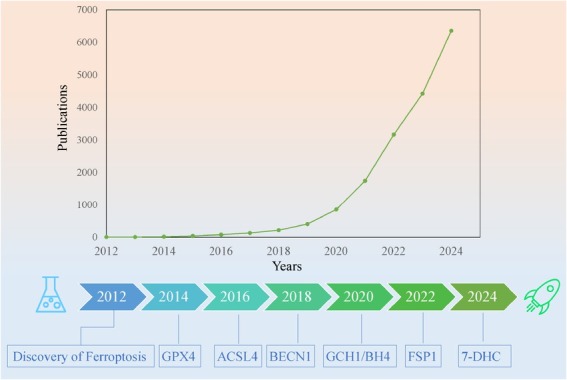
Timeline of ferroptosis and publication trends. The upper part of the figure outlines the timeline of significant discoveries of ferroptosis. The lower graph depicts the number of published articles indexed in the PubMed database under the keyword “Ferroptosis”. The concept of ferroptosis was first introduced in 2012;^[30]^ glutathione peroxidase 4 (GPX4) was identified as a key inhibitor of ferroptosis in 2014;^[31]^ the mechanism by which acyl-CoA synthetase long chain family member 4 (ACSL4) induces ferroptosis was elucidated in 2016;^[32]^ BECN1 was shown to inhibit the functionality of solute carrier family 7 member 11 (SLC7A11) in 2018;^[33]^ GPX4-independent anti-ferroptotic system involving guanosine triphosphate cyclohydrolase-1 (GCH1)/BH4 (tetrahydrobiopterin) was discovered in 2020;^[34]^ Nuclear factor erythroid 2-related factor 2 (NRF2) was found to transcribe ferroptosis suppressor protein 1 (FSP1), promoting resistance to ferroptosis in 2022;^[35]^ 7-dehydrocholesterol (7-DHC) was identified as an endogenous inhibitor of ferroptosis in 2024.^[36]^

Ferroptosis, an emerging and novel mechanism of cell death, was discovered and defined in 2012, a programmed cell death driven by iron-dependent lipid peroxidation. This may provide new therapeutic opportunities against cancers refractory to conventional therapies, and is considered a new cancer treatment strategy. It is also an important mechanisms in cancer therapies, such as radiotherapy, immunotherapy, chemotherapy, and targeted therapy.^[27]^ This concept has aroused great interest among researchers, and numerous studies have successively discovered distinct ferroptosis regulatory mechanisms and key modulators (Figure 1). The corresponding literature reports are increasing annually, with approximately 7000–9000 ferroptosis-related studies likely to be published in 2025.^[28,29]^ However, the potential interactions between ferroptosis and immunotherapy in breast cancer, as well as pharmacological options targeting ferroptosis in breast cancer, remain poorly understood.

## Overview of ferroptosis

**Figure 2:**
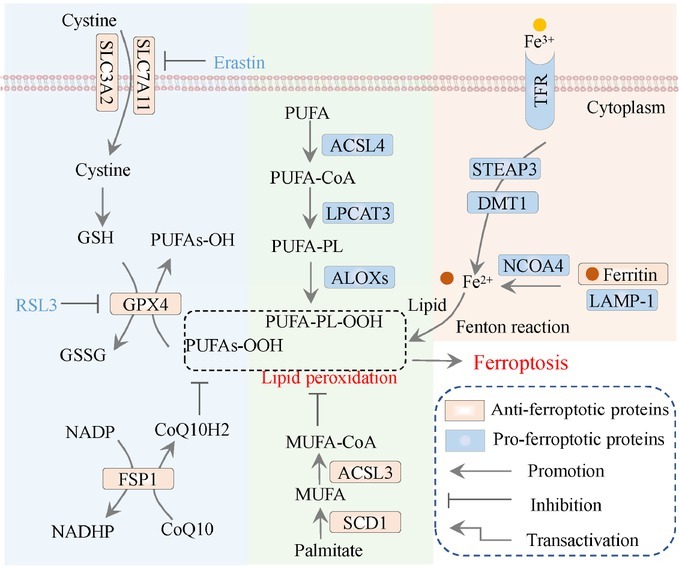
The core pathways of ferroptosis. The antioxidant mechanisms (filled by blue), lipid metabolism (filled by green), and iron metabolism (filled by orange). GSH: glutathione; GSSG: oxidized glutathione; Fe: iron ions; NO: nitric oxide. PUFA: polyunsaturated fatty acids; PUFA-CoA: PUFA with acetyl-CoA; PUFA-PL: phospholipid containing polyunsaturated fatty acid chain; PUFA-PL-OOH: PUFA phospholipid hydroperoxides; MUFA: monounsaturated fatty acyl; MUFA-CoA: MUFA with acetyl-CoA; CoQ10: Coenzyme Q10/ubiquinone; CoQ10H2: reduced CoQ10/Ubiquinol.

**Figure 3:**
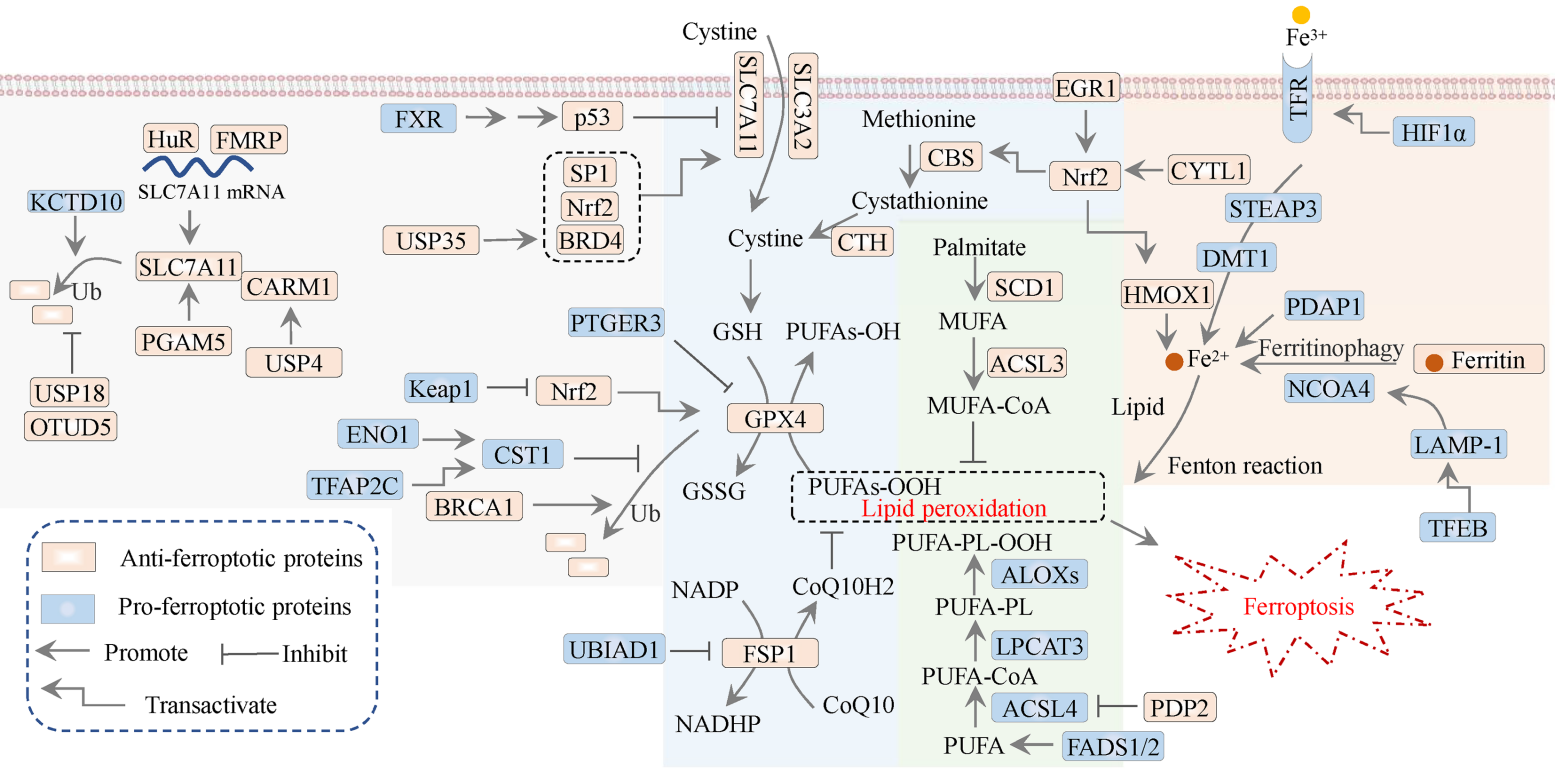
Signaling modulation of ferroptosis in breast cancer. The antioxidant mechanisms (filled by blue), lipid metabolism (filled by green), iron metabolism (filled by orange), and the regulatory mechanisms of SLC7A11/GPX4 axis (filled by gray) in breast cancer. CTH: cystathionine γ-lyase; SLC3A2, solute carrier family 3 member 2; ALOX: lipoxygenase; USP: ubiquitin-specific protease; Nrf2, nuclear factor erythroid 2-related factor 2; FSP1: ferroptosis suppressor protein 1; DMT1: divalent metal transporter 1; STEAP3: six-transmembrane epithelial antigen of prostate 3; LPCAT3: lysophosphatidylcholine acyltransferase 3; HMOX1/HO-1: heme oxygenase 1; HIF1α: hypoxia-inducible factor-1; Ub: ubiquitination.

Stockwell *et al*. first proposed the concept of ferroptosis in 2012, defining it as a type of programmed cell death characterized by the accumulation of iron-dependent lipid peroxides, distinct from apoptosis.^[30]^ The triggering of ferroptosis is critically linked to iron ions, which drive the peroxidation of polyunsaturated fatty acids (PUFAs). The overloading and buffering capacity of ferroptotic defense mechanisms will cuase the lethal accumulation of lipid peroxides in cellular membranes resulting in subsequent membrane rupture and cell death as ferroptosis.^[27]^ Ferroptosis is morphologically, biochemically, and genetically distinct from apoptosis, necrosis, and autophagy. Unique with multiple forms of regulated cell death, ferroptosis does not require caspases (mediators of apoptosis and pyroptosis), ATP depletion or mitochondrial reactive active oxygen (ROS) generation (mediators of necrosis), Bax/Bak (regulators of the mitochondrial membrane potential), or elevated intracellular Ca^2+^ level. Consequently, a combination of specific biomarkers can differentiate ferroptosis from other modes of cell death, including but not limited to lipid peroxides, mitochondrial morphology, and gene expression (*e.g*., SLC7A11 and ACSL4).^[38]^ The mechanisms that modulate or trigger ferroptosis involve lipid metabolism, antioxidant defense systems, and iron metabolism (Figure 2). Here, we collected recent studies involving the above three ferroptotic signaling pathways to shed light on the detailed molecular modulation of ferroptosis in breast cancer (Figure 3).

### Lipid metabolism

Lipid peroxidation is a free radical-driven reaction that involves unsaturated fatty acids within the cell membrane. The products of lipid peroxidation include initial lipid peroxides (LOOHs) and subsequent reactive aldehydes, such as malondialdehyde (MDA) and 4-hydroxynonenal (4-HNE). Fatty acids are categorized into three types: saturated fatty acids with no double bonds, monounsaturated fatty acids (MUFA), and PUFA that characterized by multiple double bonds.^[39]^ ACSL4 and LPCAT3 were the first proferroptotic factors identified to be involved in the activation of PUFAs particularly represented by arachidonic acid (AA),^[38]^ and their incorporation into cellular membrane lipids.^[32]^ Furthermore, ACSL4 drives ferroptosis through the accumulation of oxidized phospholipid components, particularly phosphatidylethanolamine (PE).^[32]^ Unlike the classic ferroptosis driver, PUFAs, diacyl-PUFA phosphatidylcholines (PC-PUFA) have also been reported to induce ferroptosis, which interacts with the mitochondrial electron transport chain to generate ROS that initiate lipid peroxidation and subsequently induce ferroptosis.^[40]^ The metabolism of PUFAs driven by ACSL4 increases cell sensitivity to ferroptosis, whereas ACSL3-dependent MUFA metabolism inhibits ferroptosis by competing with PUFAs for binding with membrane phospholipids.^[41]^ E-cadherin upregulates the expression of ACSL4 through the Merlin-Hippo-YAP signaling pathway, thereby indirectly influencing ferroptosis sensitivity.^[42]^

Owing to the metabolic abnormalities of PUFAs in basal-like breast tumors, targeting polar metabolomics to induce ferroptosis has been regarded as a potential therapeutic strategy.^[43]^ Recently, fatty acid desaturases 1 and 2 (FADS1/2) have been shown to modulate ferroptosis sensitivity by facilitating PUFA biosynthesis in TNBC.^[44]^ In addition, SBFI26 upregulates the ferroptosis-driving factor HMOX1 and ALOX12 to accelerate lipid peroxidation in TNBC cells.^[45]^ Interestingly, the long non-coding RNA (lncRNA) Lnc-FASA increases the sensitivity and susceptibility of TNBC cells by inhibiting the peroxidase activity of the anti-ferroptotic protein PRDX1 in a liquid-liquid phase separation manner.^[46]^ The phosphatase pyruvate dehydrogenase phosphatase 2 (PDP2) dephosphorylates ACSL4 to suppress ferroptosis in the luminal-A breast cancer.^[47]^

### Ferroptosis defense systems

There are three cellular defense mechanisms (antioxidant mechanisms) against ferroptosis: the SLC7A11/GSH/ GPX4 system, FSP1/CoQH2 (ubiquinol) signaling, and GCH1/BH4 axis, and all of them are based on the elimination of ROS and lipid peroxides to abrogate ferroptosis.^[48]^ The SLC7A11/GSH/GPX4 axis is the most extensively studied and established anti-ferroptotic signaling pathway. GSH functions as a crucial cofactor for the activity of GPX4 enzyme in eliminating ROS, whereas cysteine is the rate-limiting precursor required for GSH synthesis.^[49]^ Generally, cancer cells take up extracellular cysteine through the cystine-glutamate antiporter system xc-, which facilitates the uptake of cystine (the oxidized dimer of cysteine). In this process, SLC7A11 serves as one of the two transport protein subunits of system xc-, participating in the transmembrane transport of cystine.^[50]^ GPX4 catalyzes the reduction of lipid peroxides, including polyunsaturated fatty acid peroxides (PUFA-OOH) and phospholipid peroxides (PL-OOH)^[51]^ and converts them into their corresponding lipid alcohols. This prevents the accumulation of lipid peroxides, thereby averting membrane rupture and the subsequent onset of cellular ferroptosis.^[27]^ Although the SLC7A11-GSH-GPX4 axis is thought to be a critical mechanism for intracellular ferroptosis defense, certain cancer cell lines remain resistant to ferroptosis even after GPX4 inactivation,^[52]^ indicating the existence of alternative defense mechanisms that are GPX4-independent.^[48]^

BRCA1, a gene with prevalent mutation in breast cancer, promotes GPX4 ubiquitination and degradation; a GPX4 inhibitor exhibits synergistic efficacy against breast cancer together with a BRCA inhibitor.^[53,54]^ USP11 stabilizes PGAM5 by preventing PGAM5 ubiquitination, and PGAM5 inhibits ferroptosis *via* SLC7A11/GPX4 and Keap1/Nrf2 axis.^[55]^ Prostaglandin E receptor 3 (PTGER3), a tumor suppressor in TNBC, downregulates GPX4 and PI3K-AKT axis to enhance ferroptosis sensitivity.^[56]^ KCTD10 and USP 18 are responsible for the ubiquitination-dependent degradation and de-ubiquitination-dependent stability of SLC7A11, respectively.^[57]^ Likewise, the deubiquitinase OTUD5, which is inhibited by paclitaxel, stabilizes SLC7A11 through deubiquitination, maintaining paclitaxel resistance in TNBC.^[58]^ USP4 deubiquitinates CARM1 to maintain CARM1-mediated SLC7A11 stability.^[59]^ The deubiquitinase USP35 inhibits ferroptosis *via* deubiquitinating and stabilizing bromodomain protein 4 (BRD4): the “reader” of histone acetylation, upregulating SLC7A11 in ER+ breast cancer;^[60]^ BRD4 inhibition also decreases GPX4 protein expression.^[61]^ Interestingly, Nrf2/SLC7A11 and p53/SLC7A11 are negatively regulated by theWEE1/SETDB1/Histone 3 lysine 9 trimethylation (H3K9me3, a transcription blockade marker in epigenetics) axis.^[62,63]^

Acidosis, a hallmark of the tumor microenvironment, causes ferroptosis in breast cancer cells in a ZFAND5/SLC3A2-dependent manner. Additionally, it induces an adverse TME through promoting M1 macrophage polarization in breast cancer.^[64]^ Inhibition of CDK4/6 suppresses the transcription factor SP1 to decrease SLC7A11 expression in luminal-A breast cancer cells.^[65]^ Suppression of GPX4 enhances the sensitivity of ER+ breast cancer cells to the CDK4/6 inhibitor (palbociclib) by promoting the peroxisome AGPAT3, instead of ACSL4.^[66]^ Additionally, the RNA binding protein fragile X mental retardation protein (FMRP) prevents ferroptosis in breast cancer cells and maintains the mRNA stability of SLC7A11 *via* mediating m6A modification and interacting with splicing factor hnRNPM.^[67,69]^ Another RNA-binding protein, Hu antigen R (HuR), stabilizes SLC7A11 mRNA; its inhibition mediated by the small molecule inhibitor KH-3 triggers ferroptosis in breast cancer cells.^[70]^ Additionally, the RNA-binding protein ILF3 augments CEP55 mRNA stability to upregulate SLC7A11 and GPX4;^[11]^ however, more evidence is needed to decipher how CEP55 influences SLC7A11/GPX4 in cancer biology. Interestingly, LRP8 (ApoER2) facilitates ferroptotic resistance through promoting the translation of GPX4 in a ribosome-dependent manner.^[72]^

Downregulation of cytokine-like protein 1 (CYTL1) observed in breast cancer tumor tissues, promotes the biosynthesis of anti-ferroptotic metabolite cysteine through the Nrf2/Keap1/cystathionine β-synthase (CBS) pathway axis.^[73]^ EGR1, a tumor suppressor downregulated in breast cancer, promotes erastin-induced ferroptosis by activating the Nrf2-HMOX1 signaling axis.^[74]^ Conversely, the oncogenic MAGEA6 facilitates the AMPK/SLC7A11 axis to improve the sensitivity of TNBC cells to doxorubicin.^[75]^ HMGB3, abnormally expressed in various solid carcinomas, upregulates the expression of anti-ferroptotic proteins (SLC7A11, GPX4, and SLC3A2).^[76]^ In addition, the CoQ10 synthase UBIAD1 (COQ2) enhances ferroptotic sensitivity by decreasing GPX4 and FSP1 expression in breast cancer cells.^[77]^ The farnesoid X receptor (FXR), a scavenger of lipid peroxidation, competitively prevents CREB binding protein (CBP) from binding p53 and subsequent p53 acetylation, which attenuates ferroptosis sensitivity by downregulating the p53/SLC7A11 axis.^[78]^ Cystatin SN (CST1) maintains GPX4 activity *via* inhibiting GPX4 ubiquitination;^[79]^ FLT3 is reported as a ferroptosis suppressor;^[80]^ while TFAP2C transactivates CST1 and FLT3 to block ferroptosis.^[81]^ The glycolytic enzyme enolase 1 (ENO1) positively regulates CST1 and activates mTOR signaling, connecting glycolysis and ferroptosis.^[82]^ Similarly, a phenomenon involving SLC12A5 mediating the crosstalk between glucose metabolism and ferroptosis is also observed.^[83]^ Another research involving glycolytic the enzyme IDH2 shows that SENP1 stabilizes SIRT3 through de-SUMOylation, maintaining the anti-ferroptotic activity of the SIRT3/IDH2/GSH.^[84]^ Given the crucial role of crosstalk between cellular metabolism and ferroptosis in carcinomas,^[85]^ we look forward to further studies focusing on this direction.

### Iron metabolism

Iron metabolism is fundamental to the process of ferroptosis; it encompasses the uptake, utilization, storage, and efflux of iron ions.^[86]^ Iron ions are internalized into cells *via* the binding of transferrin (iron-containing transferrin, TF) to transferrin receptors (TFR/TFRC), followed by endocytosis of the transferrin complex.^[87]^ Regarding iron storage, various cellular processes regulate the cell susceptibility to ferroptosis by modulating the labile iron pool (LIP). An increase in the intracellular LIP can directly or indirectly generate ROS, leading to the lipid peroxidation and eventually triggering ferroptosis.^[88]^ Additionally, the intracellular LIP can produce free radicals, such as hydroxyl radicals, through Fenton reactions, contributing to the peroxidation of phospholipids.^[89]^

Autophagy also promote ferroptosis by degrading ferritin, an iron storage protein in cancer cells, which is defined as ferritinophagy.^[90]^ Thus, the autophagic modulators nuclear receptor coactivator 4 (NCOA4) and glutamate oxaloacetate transaminase 1 (GOT1) can increase or decrease LIP in an autophagy-dependent manner.^[86,91]^ Hypoxia-inducible factor 1-alpha (HIF1α) is the upstream transcriptional factor of TFRC, connecting ferroptosis and chemoresistance in breast cancer.^[92]^ ERBB2-driven cell motility 1 (MEMO1) exhibits a pattern similar to that of iron-containing extradiol dioxygenases in terms of binding and carrying iron, suggesting that MEMO1 is a potential iron transporter in breast cancer.^[93]^ Pro-ferroptotic role of PDGFA-associated protein 1(PDAP1) is associated with elevated lysosomal autophagy.^[94]^

### Regulation of ferroptosis

Several transcription factors (P53, NFE2L2/NRF2, ATF3, ATF4, YAP 1, and HIF1α) modulate ferroptosis sensitivity by translationally regulating various ferroptosis-promoting or ferroptosis-suppressing proteins.^[95]^ Additionally, epigenetic mechanisms influence ferroptosis sensitivity by affecting transcription factor activity and post-transcriptional regulatory mechanisms, including chromatin remodeling, non-coding RNAs, histone modifications, and DNA methylation.^[96]^

Interestingly, ferroptosis is considered to be autophagy-dependent. The autophagy receptor SQSTM1/p62 facilitates degradation of the iron export protein ferroportin 1 (FPN1) in lysosomes, leading to the accumulation of intracellular iron ions, which promotes ferroptosis.^[95]^ Moreover, RAB7A-mediated lipophagy enhances lipid peroxidation in iron-deficient anemia.^[97]^ The inhibition of SLC7A11 mediated by BECN1 results in GSH depletion and subsequent ferroptosis. The expression and release of cathepsin B (CTSB) mediated by STAT3 facilitates lysosome-dependent ferroptosis, while HSP90 enhances the stability of LAMP2A, which supports the degradation of GPX4.^[98]^

Targeting oncogenic signaling pathways in TNBC, such as the PI3K-AKT, MAPK, and androgen receptor (AR) pathways, has been approved for breast cancer treatment and is under clinical development.^[99]^ The Hippo-YAP, AMP-activated protein kinase (AMPK), HIF1α, and mechanistic target of rapamycin complex 1 (mTORC1) signaling pathways regulate ferroptosis through various mechanisms.^[86,100]^ It is noteworthy that different organelles, such as the mitochondria and the endoplasmic reticulum, regulate ferroptosis in an organelle-dependent manner. For example, ROS produced by mitochondrial metabolism can drive lipid peroxidation. Additionally, mitochondria can modulate iron ion metabolism and CoQ10 synthesis, thereby interfering with ferroptosis.^[101]^ Over-activation of AKT maintains TRPML1/ARL8B-mediated lysosomal exocytosis, which reduces ferrous iron overload and membrane damage, and is involved in ferroptotic resistance.^[102]^ Given the high levels of iron and lipid metabolism in TNBC tumors, the induction of ferroptosis is a promising therapeutic strategy worth exploring.^[103]^ The crosstalk between oncogenic pathways and ferroptosis and its therapeutic potential are worth investigating.

## Ferroptosis inducers in breast cancer

The mechanisms by which certain clinically used medicines to trogger ferroptosis in tumor cells have been extensively studied. This is relevant to multiple ferroptotic proteins, such as SLC7A11 (fenbendazole, propofol), GPX4 (propofol, flubendazole), transferrin receptor (TfR) (falnidamol), ferritin heavy/light chain (FTHL1/ FTL) (artesunate), and ACSL4 (propofol). Furthermore, ferroptosis has been thought to be a promising strategy in overcoming chemotherapy resistance.^[104]^ Additionally, natural bioactive compounds also modulate NCOA4 (*e.g*., dihydroartemisinin and ursolic acid), influencing ferroptosis.^[104]^

Inhibiting the HSPA5/GPX4 pathway can enhance the sensitivity to gemcitabine in pancreatic cancer.^[105]^ Likewise, pharmacological inhibition of SLC7A11 induces ferroptosis in pancreatic cancer cells, thereby enhancing the cytotoxic effects of gemcitabine and cisplatin.^[106]^ Sorafenib, a tyrosine kinase inhibitor commonly used to treat advanced renal cell carcinoma, hepatocellular carcinoma, and thyroid cancer, causes ferroptosis in a range of cancer cells by suppressing SLC7A11.^[104]^ At the same time, sorafenib also leads to GSH depletion and accumulation of lipid ROS.^[107]^ Specifically, the p62-Keap1-NRF2 pathway regulates iron metabolism and lipid peroxidation by transcriptionally activating the expression of NQO1, HO1, and FTH1, conferring cancer cells ferroptosis resistance. Inhibiting the p62-Keap1-NRF2 pathway significantly enhances the anti-liver cancer activity of erastin and sorafenib, both *in vitro* and *in vivo*.^[108]^ Recent studies have found that the chemotherapeutic drug imetelstat, used for treating acute myeloid leukemia, triggers ferroptosis through FADS2 and ACSL4-dependent lipid peroxidation metabolism.^[109]^

**Figure 4:**
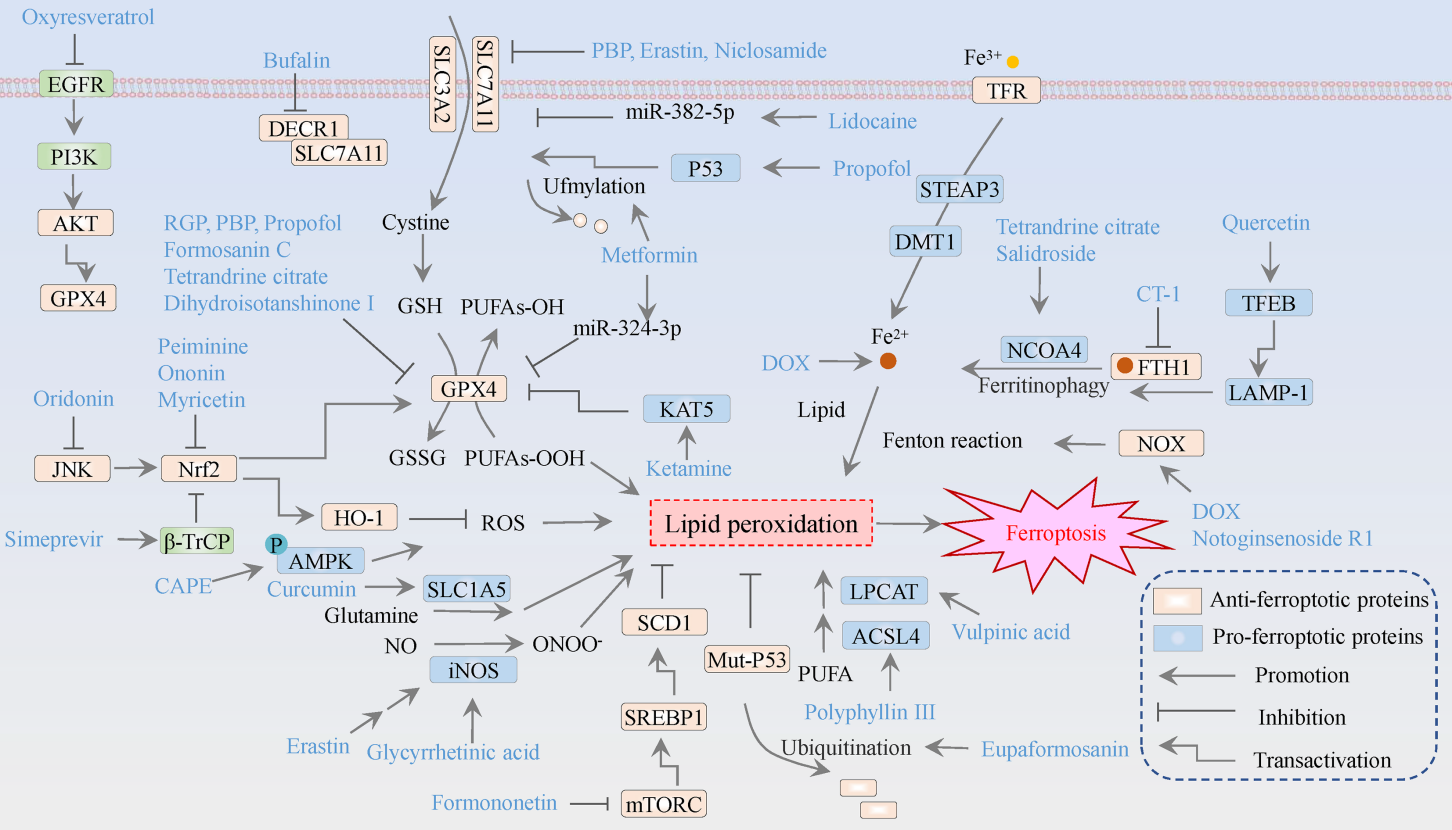
The molecular mechanisms of ferroptosis inducers against breast cancer. The orange means ferroptosis-inhibitory factor/proteins, while the blue represents ferroptosis-driving proteins. GSH refers to glutathione; GSSG denotes oxidized glutathione; Fe signifies iron ions; NO indicatesnitric oxide; ONOO^-^represents peroxynitrite; and PUFA stands for polyunsaturated fatty acids.

**Figure 5:**
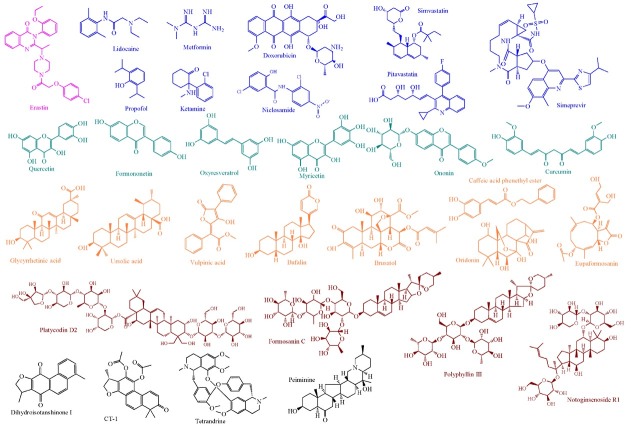
The molecular structures of representative ferroptosis inducers in breast cancer. Pink represents the ferroptosis-inducing compound: Erastin; blue indicates clinically approved drugs; light green denotes natural products: flavonoids; orange highlights natural products: acid or ester; brown exhibits saponins derived from plants; gray signifies unclassified natural products.

There is an emerging trend that a variety of small-molecule drugs and natural products function as potential ferroptotic inducers, offering novel therapeutic avenues for breast cancer (Figure 4). The underlying molecular mechanisms predominantly rely on the suppression of SLC7A11/ GPX4 signaling pathway, which drives the accumulation of lipid peroxides and enhances intracellular iron ion concentrations by interfering with iron metabolism (Figure 5). Several specific cases of breast cancer are as follows. An intriguing study revealed that erastin induces ferroptosis in MDA-MB-231 cells *via* the GSH/PDI/NO/ROS axis, leading to the production of lipid peroxidation in a manner independent of GPX4.^[110]^ In breast cancer MCF-7 cells, quercetin promotes the degradation of the iron storage protein ferritin through the autophagic TFEB/LAMP-1 axis, resulting in elevated intracellular iron ion levels and subsequent ferroptosis.^[111]^ A similar mechanism has been observed with phenazine derivatives in breast cancer cells, involving the upregulation of iron storage and transport proteins, such as IRP2, TfR1, and ferritin, resulting in iron overload.^[112]^ In addition, dihydroisotanshinone I induces ferroptosis in MCF-7 and MDA-MB-231 cells by downregulating GPX4.^[113]^ The pro-ferroptotic property of tanshinone IIA depends on its bioactivity in inhibiting the KDM1A/PIAS4/SLC7A11 axis; suppressed PIAS4 deactivates the SUMOylation of SLC7A11, in turn leading to SLC7A11 inactivation.^[114]^ However, the research doesn’t provide any evidence for the alteration of the above histone modification mediated by the histone lysine demethylase LSD1/KDM1A. The bufalin targeting 2,4-dienoyl-CoA reductase (DECR1) relieves the coordination between DECR1 and SLC7A11/GPX4 axis.^[115]^

Tetrandrine citrate promotes ferroptosis by inhibiting GPX4 and activating NCOA4-mediated ferritin phagocytosis in breast cancer cells.^[116]^ Red ginseng polysaccharide induces ferroptosis in lung cancer A549 and breast cancer MDA-MB-231 cells by downregulating GPX4,^[117]^ whereas lycium barbarum polysaccharide induces ferroptosis in MDA-MB-231 and MCF-7 cells through the downregulation of SLC7A11 and GPX4.^[118]^ Formosanin C, a saponin, has been identified as an effective ferroptosis inducer, characterized by the accumulation of lipid ROS and deprivation of GPX4 in MDA-MB-231 cells.^[119]^ The pro-ferroptotic activity of salidroside and CT-1 (a derivative of cryptotanshinone; targeting and reducing FTH1) depends on NCOA4/FTH1-mediated ferritinophagy.^[120,121]^ The terpenoids oridonin orchestrates RSL3 to induce ferroptosis in breast cancer cells *via* repressing the JNK/Nrf2/HO-1 pathway.^[122]^ Similarly, peiminine induces ferroptosis through suppressing the Nrf2/HO-1/NQO1 axis,^[123]^ while ursolic acid inhibits Keap1/Nrf2 axis to induce ferroptosis in breast cancer.^[124]^ The pro-ferroptosis effect of Paris saponin VII and myricetin involves downregulation of the Nrf2/GPX4 axis.^[125,126]^ The triterpenoid saponin, Platycodin D2, induces ferroptosis mediated by mitochondrial ROS and autophagic inhibition.^[127]^

The clinical anesthetic drug propofol (propofol) induces ferroptosis in MDA-MB-231 cells by modulating the P53/SLC7A11/GPX4 pathway.^[128]^ Another anesthetic, ketamine, induces ferroptosis in breast cancer MCF-7 and T47D cells by inhibiting the KAT5/GPX4 axis.^[129]^ The local anesthetic lidocaine facilitates ferroptosis in breast cancer T47D cells through the miR-382-5p/SLC7A11 axis.^[130]^ Moreover, antidiabetic drug metformin induces ferroptosis by inhibiting lncRNA H19-mediated autophagy.^[131]^ Another study has reported that metformin-induced ferroptosis in breast cancer cells is associated with the downregulation of SLC7A11 through ubiquitin-like modification (UFMylation).^[132]^ Metformin also promotes ferroptosis in MDA-MB-231 cells by targeting the miR-324-3p/GPX4 axis.^[133]^ Additionally, chemotherapeutic agent doxorubicin (DOX, or adriamycin) leads to ferroptosis in breast cancer cells, whereas DNAJC12 attenuates above effect *via* activating PI3K-AKT pathway to facilitate DOX-involved chemoresistance;^[134]^ DOX also activates nicotinamide adenine dinucleotide phosphate oxidase (NOX) to promote hydrogen peroxide (H2O2)/Fenton reaction in breast cancer cells;^[135]^ the effect of ferroptosis induction of Notoginsenoside R1 also depends on the activation of NOX1.^[136]^ Pharmacological activation of GPX4, however, alleviates DOX-induced heart damage and cardiotoxicity.^[137]^ In TNBC cells, the statins drug (simvastatin) weakens the HMGCR/MVA/GPX4 axis to initiate ferroptosis,^[138]^ while pitavastatin induces autophagy-dependent ferroptosis.^[139]^ The phase II clinical trial to estimate the effectiveness of simvastatin with NAC is currently underway (NCT05550415).^[140]^ Simeprevir, the agent against hepatitis C infection, induces ferroptosis *via* modulating the β-TrCP/Nrf2/GPX4 axis.^[141]^ Niclosamide, an antiparasitic medicine, decreases the expression of glutamine transporters SLC38A5 and SLC7A11 to induce ferroptosis in TNBC cells.^[142]^ Ononin, a flavonoid, induces ferroptosis *via* suppressing the Nrf2/SLC7A11 axis.^[143]^ The flavone oxyresveratrol leads to ferroptosis through inhibiting the EGFR/PI3K/AKT/GPX4 pathway axis in breast cancer.^[144]^

Curcumin increases the expression of the glutamine transporter SLC1A5 in MDA-MB-453 and MCF-7 cells to regulate the SLC1A5/GLS2/GOT1 pathway, leading to elevated lipid peroxides and subsequent ferroptosis.^[145]^ In contrast, in lung cancer cells, curcumin induces ferroptosis by upregulating ACSL4 and downregulating the SLC7A11/GPX4 axis.^[146]^ Besides, vulpinic acid, a metabolite of lichen, induces lipid peroxidation-driven ferroptosis through upregulating LPCAT3 in luminal A breast cancer cells.^[147]^ Conjugated linoleic acid triggers ferroptosis in breast cancer through ACSL1-mediated lipid peroxidation, independent of GPX4.^[148]^ Glycyrrhetinic acid induces ROS/RNS-mediated ferroptosis in breast cancer MDA-MB-231 cells by activating NADPH oxidase and iNOS while depleting GSH levels.^[149]^ Phenylethyl caffeate (CAPE) activates AMPK to induce ferroptosis in TNBC cells.^[150,151]^ In addition, polyphyllin III induces ferroptosis in MDA-MB-231 cells by upregulating ACSL4, resulting in lipid peroxide accumulation.^[152]^ Eupaformosanin promotes the ubiquitin-mediated degradation of mutant P53, leading to the accumulation of lipid reactive oxygen species and iron ions, ultimately triggering ferroptosis in triple-negative breast cancer cells.^[153]^ Formononetin, an isoflavone and FGFR2 inhibitor, induces ferroptosis *via* regulating the mTORC1/SREBP1/SCD1 axis.^[154]^

## Crosstalk between ferroptosis and tumor immune microenvironment

Immunotherapy, a novel and emerging strategy against solid cancers, has rapidly advanced in recent years, demonstrating significant effectiveness and potential for curing various carcinomas, even refractory tumors.^[155]^ Immune checkpoint inhibitors (ICIs) have been successfully implemented in cancer therapy through blocking immunosuppressive receptors, such as cytotoxic T lymphocyte antigen-4 (CTLA-4) and PD-1 in T cells, thereby enhancing the cytotoxicity and proliferative capacity of tumor-infiltrating lymphocytes (TILs). Classic ICIs include monoclonal antibodies targeting programmed cell death protein 1 (PD-1; pembrolizumab, nivolumab), programmed death ligand 1 (PD-L1, atezolizumab, durvalumab, avelumab), and CTLA-4 (ipilimumab).^[156]^ In 2019, the US Food and Drug Administration (FDA) approved the first ICI treatment regimen for breast cancer (pembrolizumab). The anti-PD-L1 antibody atezolizumab, in combination with nab-paclitaxel, was approved as a first-line treatment for PD-L1-positive metastatic TNBC patients.^[157,158]^ ICIs have demonstrated survival benefits in both advanced and early-stage TNBC patients.^[159]^ The genomic instability and mutational burden characteristic of TNBC patients lead to the generation of a greater number of neoantigens, which are recognized by the adaptive immune system, thereby stimulating an antitumor immune response. Consequently, TNBC exhibits higher levels of TILs and elevated expression of PD-L1, compared to other breast cancer subtypes. The ligand PD-L1 and its receptor PD-1 are involved in the regulation of immune tolerance. The levels of neoantigens and PD-L1 expression serve as foundational criteria for administering immunotherapy to TNBC patients.^[14,160]^ The expression status of PD-L1, the abundance of TILs, and the tumor mutational burden (TMB) are three pivotal factors influencing the response to ICI. In the context of anti-PD-1/PD-L1 therapy, PD-L1-positive TNBC patients generally exhibit improved efficacy compared to their PD-L1-negative counterparts. Conversely, patients with other subtypes of breast cancer generally exhibit lower PD-L1 expression and reduced TIL infiltration, indicating a diminished response to immunotherapeutic interventions.^[161]^ Moreover, in PD-L1-positive TNBC patients, the anti-PD-1 antibody pembrolizumab combined with chemotherapy has demonstrated improved patient survival compared to chemotherapy alone.^[162]^ In addition to its usage in combination with chemotherapy, clinical trials are underway to assess the efficacy of ICIs in conjunction with other treatments, including anti-angiogenic agents, PPAR inhibitors, and various immunotherapeutic approaches (cancer vaccines and immunomodulators).^[99]^ Notably, the efficacy of ICIs in different subtypes of TNBC remains uncertain. STAT inhibitors, cytokines or cytokine receptor antibodies, and ipilimumab (a CTLA-4 inhibitor) may be utilized for the treatment of BLIA subtype.^[17]^ In the LAR subtype of TNBC, characterized by upregulation of phosphatidylethanolamine and glutathione metabolism (particularly involving GPX4), the induction of ferroptosis through GPX4 inhibitors may enhance the efficacy of anti-PD-1 combinations.^[163]^

The small molecule compound liproxtatin-1, which inhibits lipid peroxidation of ferroptosis, significantly reduces the antitumor effects of immune checkpoint inhibitors. These findings suggest a connection and interplay between immune checkpoint blockade and ferroptosis.^[164]^ There are two approaches described in the literature to reverse therapy resistance and enhance the effectiveness of immunotherapy through the induction of ferroptosis. The first is an intrinsic method, involving the induction of ferroptosis within cancer cells to trigger a vaccine-like effect that stimulates antitumor immunity. The second is an extrinsic method, which entails to trigger ferroptosis in the tumor microenvironment to deplete immunosuppressive cells.^[165,166]^ For instance, cancer cells that undergo ferroptosis can release damage-associated molecular pattern components (DAMPs) that promote the maturation of bone marrow-derived dendritic cells.^[167]^ Additionally, ferroptotic cancer cells express specific phospholipid components that bind to toll-like receptor 2 (TLR2) on the surface of macrophages, enhancing their phagocytic activity.^[168]^ Furthermore, TGFβ1 released by macrophages promotes ferroptosis through transcriptional repression of SLC7A11 in the TGFBR/SMAD signaling pathway.^[169]^ Knockout of SLC7A11 in macrophages inhibits tumor progression and metastasis in murine liver cancer models by reducing the recruitment and infiltration of tumor-associated macrophages (TAMs), suppressing M2 polarization and enhancing the ferroptosis sensitivity of TAMs. Conversely, TNBC cell-secreted FOXM1 transactivates IDO1 to inhibit ferroptosis and promote M2 polarization of TAMs.^[170]^ Targeting SLC7A11 in TAMs using nanotechnology has been observed to enhance the antitumor efficacy of anti-PD-L1 therapies.^[171]^ Beyond immune checkpoints, co-stimulatory or co-inhibitory signals in T cells and antigen-presenting cells are also involved in the mechanisms of ferroptosis, and impact antitumor immunity;^[86]^ however, this aspect is not the primary focus of this review. The simultaneous targeting of ferroptosis and the tumor microenvironment through drug delivery/nanomedicine or biomaterials is feasible, but it is not included in our scope^.[172, 173, 174, 175]^ Here, we aim to explore the feasibility of directly modulating both PD-L1 and tumor cell ferroptosis through small molecules or key proteins (*e.g*., transcription factors and epigenetic modulators), particularly in the context of breast cancer. This approach may benefit tumor immunotherapy based on immune checkpoint inhibition, reduce the adverse effects associated with combination therapies, and even provide new alternatives for cancer pharmacotherapy.

### Ferroptosis inducers directly modulate PD-L1 expression

Fascaplysin, a natural product isolated from sponges, induces ferroptosis in lung cancer cells by promoting ROS accumulation and downregulating GPX4. Meanwhile, it upregulates PD-L1 expression, enhancing the efficacy of anti-PD-1 immunotherapy in mouse models.^[176]^ Eicosapentaenoic acid triggers ferroptosis by inhibiting the Nrf2/GPX4 pathway while concurrently suppressing IL-6/STAT3 signaling to reduce PD-L1 expression, thereby increasing sensitivity of osteosarcoma to cisplatin.^[177,178]^ Ferroptosis inducers (such as erastin and RSL3) have been reported to upregulate PD-L1 expression in TNBC cells, which may benefit immunotherapy in high-risk early TNBC patients following neoadjuvant chemotherapy.^[179]^ Additionally, in recurrent TNBC, ferroptosis inducers may exhibit potential synergistic effects with anti-PD-L1 immunotherapy.^[179]^

The upstream transcriptional factors of PD-L1 have been extensively investigated in immune evasion and clinical pharmacotherapy, such as Nrf2, STAT3.^[180,181]^ On the other hand, they have great potential for ferroptosis regulation. For instance, Nrf2 transactivates anti-ferroptotic factors involving iron metabolism (*e.g*., FTH1) and GSH metabolism (*e.g*., SLC7A11, GPX4, and PRDX1) to prevent cancer cells from ferroptosis.^[182]^ Additionally, STAT3 transcriptionally upregulates the SLC7A11/GPX4 axis and FTH1 to resist ferroptosis;^[183,184]^ STAT3 has been reported to modulate TAM recruitment and activation in a ferroptosis-dependent manner, dominating immunotherapy efficiency.^[185]^ These results suggest that targeting the common upstream ferroptosis drivers and immunosuppressive factors to induce cancer cell ferroptosis and reshape TME is promising and forthcoming. Natural products may kill two birds with one stone by targeting these transcription factors in anticancer therapy.

### Bridging ferroptosis and antitumor immunity

The CD8+ T cells derived interferon gamma (IFN-γ) inhibits the transcription of SLC7A11 and SLC3A2, which together form the cystine/ glutamate antiporter system xc-, through the activation of signal transducer and activator of transcription 1 (STAT1). This mechanism subsequently triggers ferroptosis in cancer cells.^[164]^ Similarly, radiotherapy activates ataxia telangiectasia mutated protein (ATM) to suppress the expression of SLC7A11, thereby enhancing ferroptosis sensitivity.^[186]^ Another case is that IFN-γ activates interferon receptor (IFNR)/JAK/STAT1 signaling pathway, causing the transcriptional factor interferon regulatory factor 1(IRF1) to transactivate ACSL4 expression in tumor cells. This subsequently alters the ACSL4-mediated neoplastic lipidomic.^[187]^ Notedly, the NOD-, LRR- and CARD-containing 5 (NLRC5) is activated by IFN-γ/STAT1 axis; NLRC5, the key upstream transcriptional factor of the major histocompatibility complex class I (MHC-I) family, facilitates neoantigen presentation in tumor immunity.^[188]^ Recently, ACSL promotes MHC-I-mediated antigen presentation (membrane protein involving the recognition of T cells towards tumor cells) in a NLRC5-dependent manner, enhancing anti-PD1 immunotherapy efficiency.^[189]^ These results imply that there is a potential crosstalk between ferroptotic driver ACSL4 and tumor microenvironment.

Protein kinase C (PKC), a family of protein kinases, includes eight PKC isozymes (*i.e*., PKCα, PKCβΙ, PKCβΙΙ, PKCγ, PKCδ, PKCɛ, PKCθ, and PKCη), which regulate the activity of downstream proteins by phosphorylating their serine/threonine residues.^[190]^ PKCβII, activated by ferroptosis inducer-driven lipid peroxide, phosphorylates ACSL4 at Thr328 to activate ACSL4, which triggers biosynthesis of PUFA-containing lipids. Finally, the lipid peroxidation-PKCβII-ACSL4 positive feedback axis initiates ferroptosis by promoting lipid peroxidation to amplify it to lethal levels. Targeting the PKCβII-ACSL4 axis augments the efficacy of ICI therapy.^[191]^ The further clinical study finds that ACSL4 expression is highly associated with T cell-related immune score and is a potential predictor for anti-PD1 immunotherapy response.^[191]^ Among melanoma patients, those with high ACSL4 expression have better sensitivity towards immunotherapy.^[187]^ Simultaneously, ACSL4 is regarded as an immune-related prognostic biomarker in cholangiocarcinoma.^[192]^ However, how ACSL4 influences T cell-involving antitumoral effects and underlying cell communications needs further investigation.

Androgen Receptor (AR) is expressed in over 70% of breast cancer, and its transactivation facilitates breast cancer progression in a subtype-dependent manner; targeting AR is considered an alternative strategy for treating breast cancer due to the success of AR inhibitors in prostate cancer.[193] Transnationally upregulated by AR, membrane-bound O-acyltransferase domain-containing 1 and 2 (MBOAT1/2), remodels cellular phospholipid composition to inhibit ferroptosis in breast cancer through a mechanism independent of GPX4 or FSP1.^[194]^ Naturally, higher levels of AR expression and activity were observed in male breast cancer compared with female breast cancer.^[195]^ Approximately 20%–40% of TNBC patients are of the LAR subtype, which is characterized by luminal-like gene expression, neoadjuvant chemotherapy resistance, and AR overexpression; AR directly transactivates GPX4 in LAR TNBC.[163] A series of clinical trials focuses on the treatment potential of targeted AR in AR-positive breast cancer patients,[196] including in combination with anti-PD-1 drugs.[197] Whether AR affects, however, PD-L1 expression or even immune checkpoint blockade efficacy is inconclusive.

It is well-known that cancer epigenomics reshapes anti-tumor immunity, and epigenomic therapy has been employed in breast cancer.^[198, 199, 200]^ BRD4, the “reader” of histone acetylation, facilitates permissive gene transcription;^[201,202]^ BRD4 inhibitor is a potential ferroptosis inducer in breast cancer *via* deactivating ferroptotic defense system^[203, 204, 205]^ and inducing ferritinophagy.^[61]^ Meanwhile, ACSS2, an acetyl-CoA synthetase, confers cellular proteins acetylation, including histone proteins; it also inhibits ferroptosis *via* the E2F1/SLC7A11/GPX4 axis in breast cancer brain metastatic cells.^[206]^ Additionally, the enhancer of Zeste 2 (EZH2), a subunit of polycomb repressive complex 2 (PRC2) that methylates histone H3 lysine 27 trimethylation (H3K27me3) to induce gene silencing, while AR is transactivated by EZH2 without a classic transcription-inhibitory role.[207,208] EZH2 downregulates PD-L1 expression and stability in hepatocellular and colorectal cancer, respectively.[209,210] Conversely, EZH2 positively modulates PD-L1 expression in non-small cell lung cancer.[211] On the other hand, EZH2 suppresses ferroptosis in hepatocellular cancer (downregulating transferrin receptors-2: TFR2, upregulating FSP1 and SLC7A11)[212,213] and esophageal squamous carcinoma (inhibiting ACSL4).[214] These evidences suggest EZHZ inhibition (EZH2 inhibitor, tazemetostat) is a potent strategy to realize co-targeting ferroptosis and suppressive TME (*e.g*., PD-L1-mediated immune evasion) in breast cancer.

Another promising epigenetic modulator concurrently realizes that it is protein arginine methyltransferase family (PRMT), the writers of arginine methylation consisting of nine members: PRMT1-3, CARM1(PRMT4), PRMT5-9 that induces ferroptosis and inhibits suppressive TME.^[215]^ It methylates arginine to deeply involve protein post-translational modification and histone modification-mediated gene transcription in cancers.^[216]^ Numerous studies have unveiled that PRMT1 and PRMT5 function as tumor drivers in breast cancer through methylating key proteins of onco-pathways and promoting oncogene transcription (PRMT1 confers asymmetric di-methylation of arginine (R) 3 residue of histone H4:H4R3me2a; PRMT5 catalyzes symmetric di-methylation of histone H3 arginine 2:H3R2me2s).^[217]^ PRMT1 and PRMT5 inhibitors have been introduced into phase I clinical trials for various solid tumors and hematoma.^[215,216]^ Excitingly, PRMT5 methylates GPX4 and maintains GPX4 stabilization by preventing FBW7-mediated GPX4 ubiquitination, highlighting that PRMT5 inhibition may improve ferroptotic sensitivity.^[218]^ However, a recent work exhibites PRMT1, serving as a pro-ferroptotic driver, decreases SLC7A11 transcription and inhibits FSP1 activity through methylating FSP1, which enhances ferroptosis sensitivity in acute liver injury.^[219]^ Whether PRMT can intervene ferroptosis by regulating the transcriptional expression of key ferroptotic factors in a histone modification-dependent manner remains understudied. Licochalcones have extensive anti-tumor bioactivity, involving regulation of multiple oncogenic pathways (*e.g*., EGFR/ERK, PI3K/AKT/mTOR, p38/JNK, JAK2/STAT3) and perturbation of cancerous phenotypes (proliferation, apoptosis, autophagy, and so on).^[220]^ Licochalcone A induces ferroptosis in liver cancer cells through downregulating the SLC7A11/GPX4 axis;^[221]^ it is also identified as a PRMT6 inhibitor for breast cancer treatment in preclinical research.^[222]^ However, the potential property of licochalcones to induce ferroptosis and reshape TME in a PRMT-dependent manner is poorly understood.

**Figure 6:**
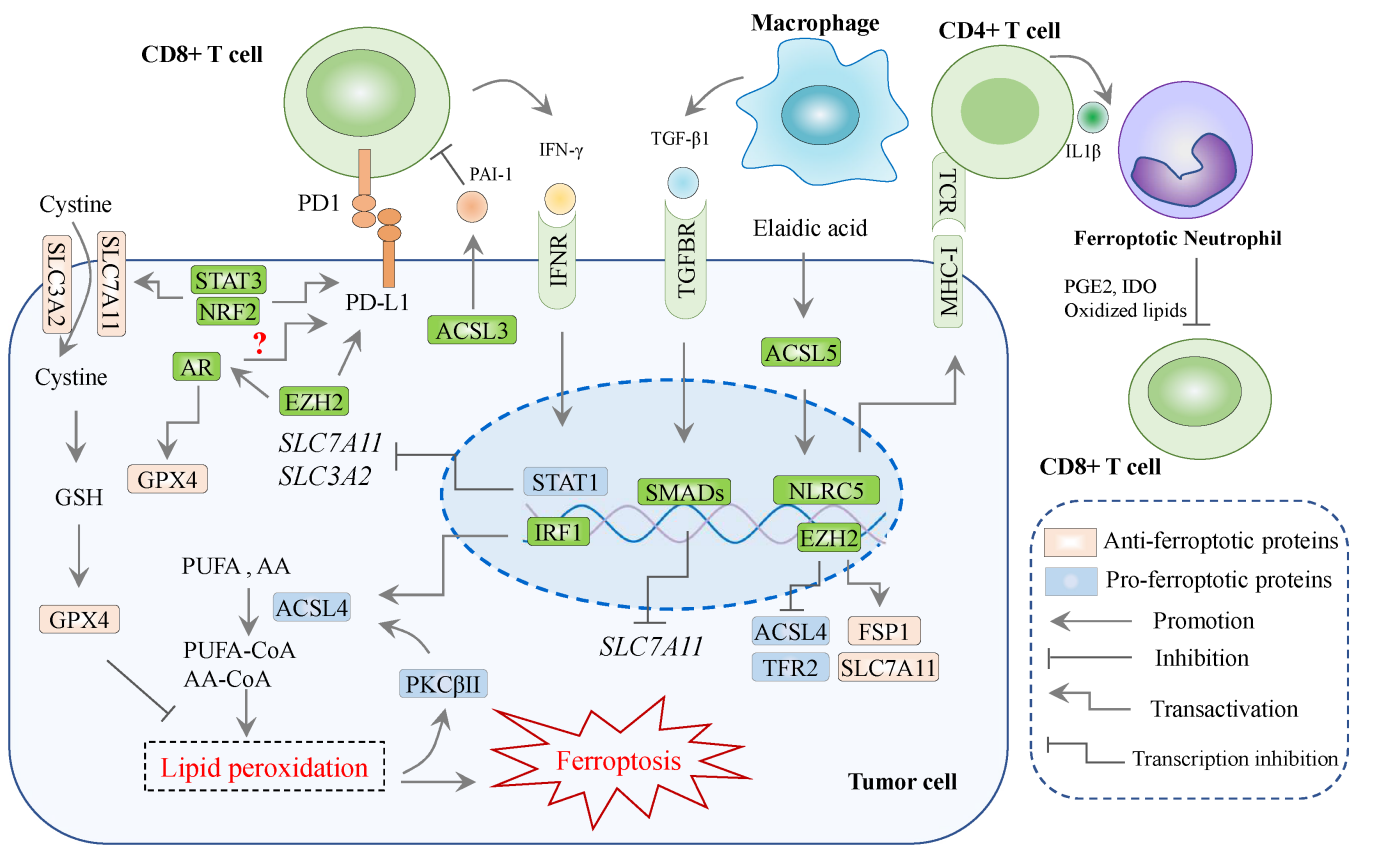
The crosstalk between ferroptosis and tumor immunity. Elaidic acid-activated ACSL5 promotes the NLRC5/MHC-I axis to enhance antigen presentation.^[189]^ TGFβ1 released by macrophages promote tumoral ferroptosis through activating transcriptional factor SMAD to inhibit SLC7A11 expression.^[169]^ CD4+ T cell-released IL1β facilitates neutrophil ferroptosis; these ferroptotic neutrophils dysfunction CD8+ T cells through generating immune-inhibiting factors.^[223]^ ACSL3 attenuates T-cell efficacy through promoting PAI-1 release.^[224]^ CD8+ T cell-driven IFN-γ promotes tumor ferroptosis through enhancing IFNR/IRF1/ACSL4^[187]^ and inhibits SLC7A11 and SLC3A2 transcription through IFNGR/JAK/STAT1.^[164]^ AA: arachidonic acid; PAI-1: plasminogen activator inhibitor type.

In brief, we expect more research that focuses on the feasibility of co-targeting ferroptosis and TME through regulating transcriptional factors or epigenomic modulators in breast cancer from bench to bedside. It is promising and reasonable to dig hit and lead compounds from natural products to achieve the above concept (Figure 6).

## Conclusion

Based on the core regulatory signals of ferroptosis, the signal regulation of ferroptosis in breast cancer mainly focuses on transcriptional regulation and degradation of ferroptosis-regulated proteins. For instance, transcription factors (Nrf2, p53, SP1) directly transactivates SLC7A11 and GPX4. BRCA1 and CST1 promote and inhibit the ubiquitination degradation of GPX4, respectively. Furthermore, natural products considered as the molecular library have great potential for further screening of potential ferroptosis inducers to combat carcinomas, including breast cancer.^[22,225]^ Given the diversity of natural product structures, flavonoids and terpene constituents (*e.g*., glycyrrhetinic acid and platycodin D2) may have better potential as ferroptosis inducers. Mechanistically, these molecules trigger tumor ferroptosis through three ferroptotic regulatory mechanisms: the antioxidant system, iron metabolism, and lipid metabolism. However, most pharmacological studies have focused only on antioxidant system (mainly on the Nrf2/GPX4 axis) and lacked insights towards how small molecules target proteins target to modulate ferroptotic signaling pathways. Importantly, ferroptosis is also considered a form of immunogenic cell death, as evidenced by the release of immune regulatory molecules known as damage-associated molecular patterns (DAMPs), such as oxidized high mobility group box 1 (HMGB1), decorin, and TLR2.^[226,227]^ Additionally, immune cell-derived cytokines such as IFN-γ modulate the ferroptosis signaling in tumor cells, thereby affecting their sensitivity to ferroptosis. These findings underscore the interplay between ferroptosis in tumor and immune cells within TME. Targeting transcription factors (*e.g*., STAT3, AR, and Nrf2) or epigenetic regulators (*e.g*., EZH2 and PRMT) could facilitate a strategy that induces ferroptosis while simultaneously inhibiting tumor-promoting immunity. Natural products and small molecule drugs may achieve the functions of co-regulating ferroptosis and TME.

Ferroptosis-regulating proteins may not simply promote or inhibit the initiation and occurrence of ferroptosis, but rather depend on the specific molecular context. For instance, activating transcription factor 4 (ATF4) is a transcription factor that activates HSPA5, which binds to GPX4 to prevent its degradation and subsequently helps cancer cells resist ferroptosis.^[105]^ Under endoplasmic reticulum stress, ATF4 directly and transcriptionally activates ferroptosis-related proteins, such as SLC7A11, HSPA5, and CHOP, to either promote or inhibit ferroptosis.^[39]^ Whether ATF4 and other members of the ATF family simultaneously regulate ferroptosis and immune checkpoints at the transcriptional level warrants further investigation. Similarly, ferroptosis occurring in anti-tumor immune cells and immunosuppressive cells is a “double-edged sword”.^[86]^ A recent study on chemoresistant TME in breast cancer showed that neutrophil ferroptosis is mediated by the IL1β/IL1R1/NF-κb/MBOAT1 signaling, and immunosuppressive factors (PGE2, IDO, and oxidized lipids) released by these dead neutrophils alleviate the antitumor effect of CD8+ T-cells.^[223]^ Conversely, CT1, a derivative of cryptotanshinone, induces ferroptosis in TNBC cells and recruits pro-tumor N2-type neutrophils to combat breast cancer.^[121]^ Therefore, when applying ferroptosis induction strategies, it is necessary to consider distinct ferroptosis sensitivity and response mechanisms among tumor cells, anti-tumor immune cells, and immunosuppressive cells, to prevent damage to positive immune cells.

**Figure 7:**
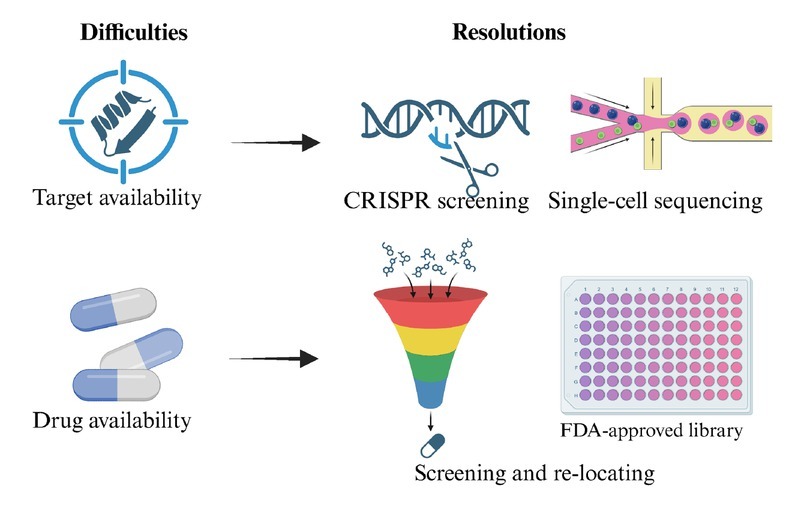
The potential challenges and resolutions of targeting the crosstalk between ferroptosis and tumor microenvironment.

This review has some limitations. The ferroptotic molecular mechanisms and clinical implications of non-coding RNAs in breast cancer are beyond our scope.^[228,229]^ Given the complex composition of extracts of medicinal plants or ethnopharmacology, we did not address them in this review.^[230]^ However, the potential contributions of Chinese herbal extracts to ferroptosis have been summarized.^[231]^ Moreover, one review has regarded ferroptosis as a therapeutic strategy for TNBC (overcoming drug resistance and combination therapy).^[140,232,234]^ Another review described the molecular mechanisms of ferroptosis in breast cancer.^[235]^ Our review provides a more comprehensive view of how natural products induce ferroptosis to combat breast cancer, compared with the previous literature.^[236]^ We underscore representative ferroptotic modulators and pathways in breast cancer, and natural products/ ferroptosis inducers against breast cancer. There are some difficulties in co-targeting ferroptosis and immune evasion:

(1) target availability, how to find novel targets mediating ferroptosis to initiate and modulate the expression of tumor immune molecules requires further practice (*e.g*., CRISPER screening, single-cell sequencing), based on the interplay of “ferroptosis-immunity” (ferroptosis-immune crosstalk). However, targeting transcription factors or epigenetic regulators is potential and promising (*e.g*., STAT3, Nrf2, AR, EZH2, PRMT); (2) drug efficiency, improvement the targeting and immune regulatory effects of small molecule drugs cannot be achieved simultaneously. The reshaping effect of small molecule drugs on TME cannot be ignored (differences in ferroptotic sensitivity between tumor cells and immune cells). Screening new lead compounds from natural products or FDA-approved drug library and studying structure-activity relationships may provide alternative therapeutic strategies (Figure 7).

The effectiveness of ferroptosis targeting has been preliminarily confirmed in a series of clinical trials (Table 1).^[237,238]^ As expected, GPX4/SLC7A11 inhibitor and ACSL4 agonist have attracted considerable interest from researchers; however, strategies targeting iron metabolism are lacking. Additionally, despite advances in immunotherapy based on immune checkpoint blockade in breast cancer, the combination of immunotherapy with other strategies (such as ferroptosis induction) is being explored to improve efficacy and maximize patient benefits.^[14]^ As forementioned, several clinical drugs have shown potential to induce ferroptosis,^[104]^ which has also been reported as an alternative strategy for reversing first-line anti-tumor drug resistance (olaparib, cetuximab, and gefitinib).^[165]^ Although ferroptosis plays a crucial role in immunotherapy, little is known about its role in breast cancer, particularly TNBC.^[239]^ Future research should focus on the epigenetic (*e.g*., histone modifications) and post-transcriptional regulations (*e.g*., protein acetylation) of ferroptosis regulators in breast cancer. Additionally, exploring the potential role of ferroptosis in the immune microenvironment could enhance therapeutic efficacy, including, but not limited to, immunotherapy. Ferroptosis inducers may represent a promising alternative strategy for breast cancer treatment. Collectively, natural products are potential ferroptosis inducers and diverse molecular scaffolds for further drug design and development. This review also lays a theoretical foundation for co-targeting the crosstalk between ferroptosis and tumor microenvironment.

**Table 1:** The clinical trials for ferroptosis-inducing strategies in cancers.

Targeted pathway	Target	Drug	Phase	Cancer	ID
Anti-oxidation metabolism	GSH	Cisplatin	III	NSCLC	NCT01656551
	DHODH	Brequinar	I, II	AML	NCT03760666
	GCL	Buthionine	I	Neuroblastoma	NCT00002730
	GCL	Sulfoximine	I	Neuroblastoma	NCT00005835
	GPX4	Altretamine	I	Lymphoma, arcoma	NCT00002936
	GPX4	Withaferin A	II	Osteosarcoma	NCT00689195
	SLC7A11	Sorafenib	II, III	HCC	NCT03794440
	SLC7A11	Sorafenib	I, II	AML	NCT03247088
	SLC7A11	Sorafenib	II	Neuroblastoma	NCT02559778
	SLC7A11	Sorafenib	II	NSCLC	NCT00064350
	SLC7A11	Sulfasalazine	N/A	Glioma	NCT01577966
	SLC7A11	Sulfasalazine	I	Glioblastoma	NCT04205357
	SLC7A11	Sulfasalazine	II	Breast cancer	NCT03847311
	SLC7A11	Temozolomide	III	Glioma	NCT00626990
	HMGCR	Fluvastatin	II	Breast cancer	NCT00416403
	HMGCR	Simvastatin	II	Breast cancer	NCT00354640
	TXNRD	Auranofin	I, II	Glioblastoma	NCT02770378
	TXNRD	Auranofin	I, II	NSCLC	NCT01737502
	TXNRD	Auranofin	II	CML	NCT01419691
	TXNRD	Auranofin	I	RPF	NCT01747798
Iron metabolism	FT	Artesunate	I	Breast cancer	NCT00764036
	Iron chelator	Ciclopirox Olamine	I	Hematologic malignancy	NCT00990587
Lipid metabolism	DRD2	Haloperidol	II	Glioblastoma	NCT06218524
	LOX	NDGA	II	Prostate cancer	NCT00678015
	ACSL4	Pioglitazone	II	Breast cancer	NCT05013255
	ACSL4	Rosiglitazone	III	Prostate cancer	NCT00182052
	ACSL4	Rosiglitazone	II	Solid tumors	NCT04114136
	ACSL4	Rosiglitazone	II	Sarcoma	NCT00004180
	ACSL4	Troglitazone	II	Sarcoma	NCT00003058
	ALOX5	Zileuton	I	CML	NCT02047149
	ALOX5	Zileuton	I	CML	NCT01130688
	ALOX5	Zileuton	II	Head and neck cancer	NCT00056004
	ALOX5	Zileuton	II	Lung cancer	NCT00070486

AML: acute myeloid leukemia; CML: chronic myelogenous leukemia; DHODH: dihydroorotate dehydrogenase; DRD2: dopamine D2 receptor; FT: ferritin; GCL: glutamate cysteine ligase; HCC: hepatocellular carcinoma; HMGCR: 3- hydroxy-3-methylglutaryl-CoA reductase; LOX: lipoxygenase; N/A: not applicable; NDGA: nordihydroguaiaretic acid; NSCLC: non-small cell lung cancer; RPF: recurrent epithelial ovarian, primary peritoneal, or fallopian tube cancer; TXNRD: thioredoxin reductase. Data from ClinicalTrals.gov.

## References

[1] Sung H, Ferlay J, Siegel RL, Laversanne M, Soerjomataram I, Jemal A (2021). Global Cancer Statistics 2020: GLOBOCAN Estimates of Incidence and Mortality Worldwide for 36 Cancers in 185 Countries. CA Cancer J Clin.

[2] Mochly-Rosen D, Das K, Grimes KV (2012). Protein kinase C, an elusive therapeutic target?. Nat Rev Drug Discov.

[3] Siegel RL, Kratzer TB, Giaquinto AN, Sung H, Jemal A (2025). Cancer statistics, 2025. CA Cancer J Clin.

[4] Siegel RL, Miller KD, Fuchs HE, Jemal A (2021). Cancer Statistics, 2021. CA Cancer J Clin.

[5] Lima SM, Kehm RD, Terry MB (2021). Global breast cancer incidence and mortality trends by region, age-groups, and fertility patterns. EClinicalMedicine.

[6] Harbeck N, Gnant M (2017). Breast cancer. Lancet.

[7] Wu S, Pan R, Lu J, Wu X, Xie J, Tang H (2022). Development and Verification of a Prognostic Ferroptosis-Related Gene Model in Triple-Negative Breast Cancer. Front Oncol.

[8] Carey L, Winer E, Viale G, Cameron D, Gianni L (2010). Triple-negative breast cancer: disease entity or title of convenience?. Nat Rev Clin Oncol.

[9] Jiang YZ, Ma D, Suo C, Shi J, Xue M, Hu X (2019). Genomic and Transcriptomic Landscape of Triple-Negative Breast Cancers: Subtypes and Treatment Strategies. Cancer Cell.

[10] Lehmann BD, Pietenpol JA (2014). Identification and use of biomarkers in treatment strategies for triple-negative breast cancer subtypes. J Pathol.

[11] Burstein MD, Tsimelzon A, Poage GM, Covington KR, Contreras A, Fuqua SA (2015). Comprehensive genomic analysis identifies novel subtypes and targets of triple-negative breast cancer. Clin Cancer Res.

[12] Robson M, Im SA, Senkus E, Xu B, Domchek SM, Masuda N (2017). Olaparib for Metastatic Breast Cancer in Patients with a Germline BRCA Mutation. N Engl J Med.

[13] Waks AG, Winer EP (2019). Breast Cancer Treatment: A Review. JAMA.

[14] Bianchini G, Balko JM, Mayer IA, Sanders ME, Gianni L (2016). Triple-negative breast cancer: challenges and opportunities of a heterogeneous disease. Nat Rev Clin Oncol.

[15] Chaudhary LN, Wilkinson KH, Kong A (2018). Triple-Negative Breast Cancer: Who Should Receive Neoadjuvant Chemotherapy?. Surg Oncol Clin N Am.

[16] Sun LI, Zhuo S, Li X, Kong H, DU W, Zhou C (2025). Astragalus polysaccharide enhances the therapeutic efficacy of cisplatin in triple-negative breast cancer through multiple mechanisms. Oncol Res.

[17] Yin L, Duan JJ, Bian XW, Yu SC (2020). Triple-negative breast cancer molecular subtyping and treatment progress. Breast Cancer Res.

[18] Shao X, Xie N, Chen Z, Wang X, Cao W, Zheng Y (2024). Inetetamab for injection in combination with vinorelbine weekly or every three weeks in HER2-positive metastatic breast cancer: A multicenter, randomized, phase II clinical trial. J Transl Int Med.

[19] Zou Y, Yang A, Chen B, Deng X, Xie J, Dai D (2024). crVDAC3 alleviates ferroptosis by impeding HSPB1 ubiquitination and confers trastuzumab deruxtecan resistance in HER2-low breast cancer. Drug Resist Updat.

[20] van den Ende NS, Nguyen AH, Jager A, Kok M, Debets R, van Deurzen CHM (2023). Triple-Negative Breast Cancer and Predictive Markers of Response to Neoadjuvant Chemotherapy: A Systematic Review. Int J Mol Sci.

[21] Song F, Tarantino P, Garrido-Castro A, Lynce F, Tolaney SM, Schlam I (2024). Immunotherapy for Early-Stage Triple Negative Breast Cancer: Is Earlier Better?. Curr Oncol Rep.

[22] Zhou J, Wang L, Peng C, Peng F (2022). Co-Targeting Tumor Angiogenesis and Immunosuppressive Tumor Microenvironment: A Perspective in Ethnopharmacology. Front Pharmacol.

[23] Ganesan K, Xu C, Wu S, Sui Y, Du B, Zhang J (2024). Ononin Inhibits Tumor Bone Metastasis and Osteoclastogenesis By Targeting Mitogen-Activated Protein Kinase Pathway in Breast Cancer. Research (Wash D C).

[24] Wang Z, Liu Z, Qu J, Sun Y, Zhou W (2024). Role of natural products in tumor therapy from basic research and clinical perspectives. Acta Materia Medica.

[25] Zhou J, Wan F, Xiao B, Li X, Peng C, Peng FU (2024). Metochalcone induces senescence-associated secretory phenotype via JAK2/STAT3 pathway in breast cancer. Oncol Res.

[26] Peng F, Zhu H, Meng CW, Ren YR, Dai O, Xiong L (2019). New Isoflavanes from Spatholobus suberectus and Their Cytotoxicity against Human Breast Cancer Cell Lines. Molecules.

[27] Lei G, Zhuang L, Gan B (2022). Targeting ferroptosis as a vulnerability in cancer. Nat Rev Cancer.

[28] Yan H, Talty R, Aladelokun O, Bosenberg M, Johnson CH (2023). Ferroptosis in colorectal cancer: a future target?. Br J Cancer.

[29] Ruan D, Wen J, Fang F, Lei Y, Zhao Z, Miao Y (2023). Ferroptosis in epithelial ovarian cancer: a burgeoning target with extraordinary therapeutic potential. Cell Death Discov.

[30] Dixon SJ, Lemberg KM, Lamprecht MR, Skouta R, Zaitsev EM, Gleason CE (2012). Ferroptosis: an iron-dependent form of nonapoptotic cell death. Cell.

[31] Yang WS, SriRamaratnam R, Welsch ME, Shimada K, Skouta R, Viswanathan VS (2014). Regulation of ferroptotic cancer cell death by GPX4. Cell.

[32] Doll S, Proneth B, Tyurina YY, Panzilius E, Kobayashi S, Ingold I (2017). ACSL4 dictates ferroptosis sensitivity by shaping cellular lipid composition. Nat Chem Biol.

[33] Song X, Zhu S, Chen P, Hou W, Wen Q, Liu J (2018). AMPK-Mediated BECN1 Phosphorylation Promotes Ferroptosis by Directly Blocking System X(c)(-) Activity. Curr Biol.

[34] Kraft VAN, Bezjian CT, Pfeiffer S, Ringelstetter L, Müller C, Zandkarimi F (2020). GTP Cyclohydrolase 1/Tetrahydrobiopterin Counteract Ferroptosis through Lipid Remodeling. ACS Cent Sci.

[35] Koppula P, Lei G, Zhang Y, Yan Y, Mao C, Kondiparthi L (2022). A targetable CoQ-FSP1 axis drives ferroptosis- and radiation-resistance in KEAP1 inactive lung cancers. Nat Commun.

[36] Li Y, Ran Q, Duan Q, Jin J, Wang Y, Yu L (2024). 7-Dehydrocholesterol dictates ferroptosis sensitivity. Nature.

[37] Reed JC, Pellecchia M (2012). Ironing out cell death mechanisms. Cell.

[38] Stockwell BR (2022). Ferroptosis turns 10: Emerging mechanisms, physiological functions, and therapeutic applications. Cell.

[39] Tang D, Chen X, Kang R, Kroemer G (2021). Ferroptosis: molecular mechanisms and health implications. Cell Res.

[40] Qiu B, Zandkarimi F, Bezjian CT, Reznik E, Soni RK, Gu W (2024). Phospholipids with two polyunsaturated fatty acyl tails promote ferroptosis. Cell.

[41] Magtanong L, Ko PJ, To M, Cao JY, Forcina GC, Tarangelo A (2019). Exogenous Monounsaturated Fatty Acids Promote a Ferroptosis-Resistant Cell State. Cell Chem Biol.

[42] Wu J, Minikes AM, Gao M, Bian H, Li Y, Stockwell BR, Chen ZN (2019). Intercellular interaction dictates cancer cell ferroptosis via NF2-YAP signalling. Nature.

[43] Jiang YZ, Ma D, Jin X, Xiao Y, Yu Y, Shi J (2024). Integrated multiomic profiling of breast cancer in the Chinese population reveals patient stratification and therapeutic vulnerabilities. Nat Cancer.

[44] Lorito N, Subbiani A, Smiriglia A, Bacci M, Bonechi F, Tronci L (2024). FADS 1/2 control lipid metabolism and ferroptosis susceptibility in triple-negative breast cancer. EMBO Mol Med.

[45] He G, Zhang Y, Feng Y, Chen T, Liu M, Zeng Y (2024). SBFI26 induces triple-negative breast cancer cells ferroptosis via lipid peroxidation. J Cell Mol Med.

[46] Fan X, Liu F, Wang X, Wang Y, Chen Y, Shi C (2024). LncFASA promotes cancer ferroptosis via modulating PRDX1 phase separation. Sci China Life Sci.

[47] Zhu JJ, Huang FY, Chen H, Zhang YL, Chen MH, Wu RH (2024). Autocrine phosphatase PDP2 inhibits ferroptosis by dephosphorylating ACSL4 in the Luminal A Breast Cancer. PLoS One.

[48] Zheng J, Conrad M (2020). The Metabolic Underpinnings of Ferroptosis. Cell Metab.

[49] Forman HJ, Zhang H, Rinna A (2009). Glutathione: overview of its protective roles, measurement, and biosynthesis. Mol Aspects Med.

[50] Koppula P, Zhuang L, Gan B (2021). Cystine transporter SLC7A11/xCT in cancer: ferroptosis, nutrient dependency, and cancer therapy. Protein Cell.

[51] Imai H, Nakagawa Y (2003). Biological significance of phospholipid hydroperoxide glutathione peroxidase (PHGPx, GPx4) in mammalian cells. Free Radic Biol Med.

[52] Viswanathan VS, Ryan MJ, Dhruv HD, Gill S, Eichhoff OM, Seashore-Ludlow B (2017). Dependency of a therapy-resistant state of cancer cells on a lipid peroxidase pathway. Nature.

[53] Xie X, Chen C, Wang C, Guo Y, Sun B, Tian J (2024). Targeting GPX4-mediated ferroptosis protection sensitizes BRCA1-deficient cancer cells to PARP inhibitors. Redox Biol.

[54] Lei G, Mao C, Horbath AD, Yan Y, Cai S, Yao J (2024). BRCA1-Mediated Dual Regulation of Ferroptosis Exposes a Vulnerability to GPX4 and PARP Co-Inhibition in BRCA1-Deficient Cancers. Cancer Discov.

[55] Zhang N, Wang Q, Lu Y, Wang F, He Z (2024). The deubiquitinating enzyme USP 11 regulates breast cancer progression by stabilizing PGAM5. Breast Cancer Res.

[56] Wang S, Zhang Y, Zhang D, Meng J, Che N, Zhao X (2024). PTGER3 knockdown inhibits the vulnerability of triple-negative breast cancer to ferroptosis. Cancer Sci.

[57] Zhou Q, Yu H, Chen Y, Ren J, Lu Y, Sun Y (2024). The CRL3(KCTD10) ubiquitin ligase-USP18 axis coordinately regulates cystine uptake and ferroptosis by modulating SLC7A11. Proc Natl Acad Sci U S A.

[58] Liu X, Ma Z, Jing X, Wang G, Zhao L, Zhao X (2024). The deubiquitinase OTUD5 stabilizes SLC7A11 to promote progression and reduce paclitaxel sensitivity in triple-negative breast cancer. Cancer Lett.

[59] Li X, Yan C, Yun J, Xu X, Wei H, Xu X (2025). USP4/CARM1 Axis Promotes the Malignant Transformation of Breast Cancer Cells by Upregulating SLC7A11 Expression. Clin Breast Cancer.

[60] Cao J, Wu T, Zhou T, Jiang Z, Ren Y, Yu J (2025). USP35 promotes the growth of ER positive breast cancer by inhibiting ferroptosis via BRD4-SLC7A11 axis. Commun Biol.

[61] Sui S, Zhang J, Xu S, Wang Q, Wang P, Pang D (2019). Ferritinophagy is required for the induction of ferroptosis by the bromodomain protein BRD4 inhibitor (+)-JQ1 in cancer cells. Cell Death Dis.

[62] Xiong C, Ling H, Huang Y, Dong H, Xie B, Hao Q (2025). AZD1775 synergizes with SLC7A11 inhibition to promote ferroptosis. Sci China Life Sci.

[63] Padeken J, Methot SP, Gasser SM (2022). Establishment of H3K9-methylated heterochromatin and its functions in tissue differentiation and maintenance. Nat Rev Mol Cell Biol.

[64] Xiong H, Zhai Y, Meng Y, Wu Z, Qiu A, Cai Y, Wang G, Yang L (2024). Acidosis activates breast cancer ferroptosis through ZFAND5/SLC3A2 signaling axis and elicits M1 macrophage polarization. Cancer Lett.

[65] Cui Y, Li Y, Xu Y, Liu X, Kang X, Zhu J (2024). SLC7A11 protects luminal A breast cancer cells against ferroptosis induced by CDK4/6 inhibitors. Redox Biol.

[66] Herrera-Abreu MT, Guan J, Khalid U, Ning J, Costa MR, Chan J (2024). Inhibition of GPX4 enhances CDK4/6 inhibitor and endocrine therapy activity in breast cancer. Nat Commun.

[67] Wang N, Shi B, Ding L, Zhang X, Ma X, Guo S (2024). FMRP protects breast cancer cells from ferroptosis by promoting SLC7A11 alternative splicing through interacting with hnRNPM. Redox Biol.

[68] Zhang C, Wang S, Lu X, Zhong W, Tang Y, Huang W (2024). POP1 Facilitates Proliferation in Triple-Negative Breast Cancer via m6A-Dependent Degradation of CDKN1A mRNA. Research (Wash D C).

[69] Wang J, Zhao G, Zhao Y, Zhao Z, Yang S, Zhou A (2024). N(6)-methylation in the development, diagnosis, and treatment of gastric cancer. J Transl Int Med.

[70] Wei L, Kim SH, Armaly AM, Aubé J, Xu L, Wu X (2024). RNA-binding protein HuR inhibition induces multiple programmed cell death in breast and prostate cancer. Cell Commun Signal.

[71] Chen S, Luo Y, Ruan S, Su G, Huang G (2025). RNA binding protein ILF3 increases CEP55 mRNA stability to enhance malignant potential of breast cancer cells and suppress ferroptosis. Hereditas.

[72] Li Z, Ferguson L, Deol KK, Roberts MA, Magtanong L, Hendricks JM, Mousa GA (2022). Ribosome stalling during selenoprotein translation exposes a ferroptosis vulnerability. Nat Chem Biol.

[73] Xue W, Yu Y, Yao Y, Zhou L, Huang Y, Wang Y (2024). Breast cancer cells have an increased ferroptosis risk induced by system x(c)(-) blockade after deliberately downregulating CYTL1 to mediate malignancy. Redox Biol.

[74] Lin Z, Liu Z, Pan Z, Zhang Y, Yang X, Feng Y (2024). EGR1 promotes erastin-induced ferroptosis through activating Nrf2-HMOX1 signaling pathway in breast cancer cells. J Cancer.

[75] Zhu H, Jiang CW, Zhang WL, Yang ZY, Sun G (2024). Targeting oncogenic MAGEA6 sensitizes triple negative breast cancer to doxorubicin through its autophagy and ferroptosis by stabling AMPKα1. Cell Death Discov.

[76] Luo B, Zheng H, Liang G, Luo Y, Zhang Q, Li X (2025). HMGB3 Contributes to Anti-PD-1 Resistance by Inhibiting IFN-γ-Driven Ferroptosis in TNBC. Mol Carcinog.

[77] Tosi G, Paoli A, Zuccolotto G, Turco E, Simonato M, Tosoni D (2024). Cancer cell stiffening via CoQ(10) and UBIAD1 regulates ECM signaling and ferroptosis in breast cancer. Nat Commun.

[78] Huang P, Zhao H, Dai H, Li J, Pan X, Pan W (2024). FXR deficiency induced ferroptosis via modulation of the CBP-dependent p53 acetylation to suppress breast cancer growth and metastasis. Cell Death Dis.

[79] Li D, Wang Y, Dong C, Chen T, Dong A, Ren J (2023). CST1 inhibits ferroptosis and promotes gastric cancer metastasis by regulating GPX4 protein stability via OTUB1. Oncogene.

[80] Shen J, He Y, Zhou B, Qin H, Zhang S, Huang Z (2024). TFAP2C/FLT3 axis reduces ferroptosis in breast cancer cells by inhibiting mitochondrial autophagy. Int J Biochem Cell Biol.

[81] Yuan L, Zhou D, Li W, Guan J, Li J, Xu B (2024). TFAP2C Activates CST1 Transcription to Facilitate Breast Cancer Progression and Suppress Ferroptosis. Biochem Genet.

[82] Huang G, Lu L, You Y, Li J, Zhang K (2024). Knockdown of ENO1 promotes autophagy dependent-ferroptosis and suppresses glycolysis in breast cancer cells via the regulation of CST1. Drug Dev Res.

[83] Wang H, Dai Y, Wang F (2024). ETV4 mediated transcriptional activation of SLC12A5 exacerbates ferroptosis resistance and glucose metabolism reprogramming in breast cancer cells. Mol Med Rep.

[84] Chen Y, Chen B, Hong Y, Chen L, Zheng S (2025). SENP1 promotes deacetylation of isocitrate dehydrogenase 2 to inhibit ferroptosis of breast cancer via enhancing SIRT3 stability. Biotechnol Appl Biochem.

[85] Yang S, Hu C, Chen X, Tang Y, Li J, Yang H (2024). Crosstalk between metabolism and cell death in tumorigenesis. Mol Cancer.

[86] Dang Q, Sun Z, Wang Y, Wang L, Liu Z, Han X (2022). Ferroptosis:a double-edged sword mediating immune tolerance of cancer. Cell Death Dis.

[87] Richardson DR, Ponka P (1997). The molecular mechanisms of the metabolism and transport of iron in normal and neoplastic cells. Biochim Biophys Acta.

[88] Dixon SJ, Stockwell BR (2014). The role of iron and reactive oxygen species in cell death. Nat Chem Biol.

[89] Winterbourn CC (1995). Toxicity of iron and hydrogen peroxide: the Fenton reaction. Toxicol Lett.

[90] Gao M, Monian P, Pan Q, Zhang W, Xiang J, Jiang X (2016). Ferroptosis is an autophagic cell death process. Cell Res.

[91] Hou W, Xie Y, Song X, Sun X, Lotze MT, Zeh HJ (2016). Autophagy promotes ferroptosis by degradation of ferritin. Autophagy.

[92] Yu X, Guo Q, Zhang H, Wang X, Han Y, Yang Z (2024). Hypoxia-inducible factor-1α can reverse the Adriamycin resistance of breast cancer adjuvant chemotherapy by upregulating transferrin receptor and activating ferroptosis. FASEB J.

[93] Dolgova N, Uhlemann EE, Boniecki MT, Vizeacoumar FS, Ara A, Nouri P (2024). MEMO1 binds iron and modulates iron homeostasis in cancer cells. Elife.

[94] Vinik Y, Maimon A, Dubey V, Raj H, Abramovitch I, Malitsky S (2024). Programming a Ferroptosis-to-Apoptosis Transition Landscape Revealed Ferroptosis Biomarkers and Repressors for Cancer Therapy. Adv Sci (Weinh).

[95] Li J, Liu J, Xu Y, Wu R, Chen X, Song X (2021). Tumor heterogeneity in autophagy-dependent ferroptosis. Autophagy.

[96] Freitas-Cortez MA, Masrorpour F, Jiang H, Mahmud I, Lu Y, Huang A (2025). Cancer cells avoid ferroptosis induced by immune cells via fatty acid binding proteins. Mol Cancer.

[97] Lee S, Hwang N, Seok BG, Lee S, Lee SJ, Chung SW (2023). Autophagy mediates an amplification loop during ferroptosis. Cell Death Dis.

[98] Zhou B, Liu J, Kang R, Klionsky DJ, Kroemer G, Tang D (2020). Ferroptosis is a type of autophagy-dependent cell death. Semin Cancer Biol.

[99] Bianchini G, De Angelis C, Licata L, Gianni L (2022). Treatment landscape of triple-negative breast cancer - expanded options, evolving needs. Nat Rev Clin Oncol.

[100] Dixon SJ, Pratt DA (2023). Ferroptosis: A flexible constellation of related biochemical mechanisms. Mol Cell.

[101] Dixon SJ, Olzmann JA (2024). The cell biology of ferroptosis. Nature Reviews Molecular Cell Biology.

[102] Zhang HL, Hu BX, Ye ZP, Li ZL, Liu S, Zhong WQ (2024). TRPML1 triggers ferroptosis defense and is a potential therapeutic target in AKT-hyperactivated cancer. Sci Transl Med.

[103] Xiao Y, Ma D, Yang YS, Yang F, Ding JH, Gong Y (2022). Comprehensive metabolomics expands precision medicine for triple-negative breast cancer. Cell Res.

[104] Wang Y, Wu X, Ren Z, Li Y, Zou W, Chen J (2023). Overcoming cancer chemotherapy resistance by the induction of ferroptosis. Drug Resist Updat.

[105] Zhu S, Zhang Q, Sun X, Zeh HJ, Lotze MT, Kang R (2017). HSPA5 Regulates Ferroptotic Cell Death in Cancer Cells. Cancer Res.

[106] Daher B, Parks SK, Durivault J, Cormerais Y, Baidarjad H, Tambutte E (2019). Genetic Ablation of the Cystine Transporter xCT in PDAC Cells Inhibits mTORC1, Growth, Survival, and Tumor Formation via Nutrient and Oxidative Stresses. Cancer Res.

[107] Dixon SJ, Patel DN, Welsch M, Skouta R, Lee ED, Hayano M (2014). Pharmacological inhibition of cystine-glutamate exchange induces endoplasmic reticulum stress and ferroptosis. Elife.

[108] Sun X, Ou Z, Chen R, Niu X, Chen D, Kang R (2016). Activation of the p62-Keap1-NRF2 pathway protects against ferroptosis in hepatocellular carcinoma cells. Hepatology.

[109] Bruedigam C, Porter AH, Song A, Vroeg In de Wei G, Stoll T, Straube J (2024). Imetelstat-mediated alterations in fatty acid metabolism to induce ferroptosis as a therapeutic strategy for acute myeloid leukemia. Nat Cancer.

[110] Wang H, Wang P, Zhu BT (2022). Mechanism of Erastin-Induced Ferroptosis in MDA-MB-231 Human Breast Cancer Cells: Evidence for a Critical Role of Protein Disulfide Isomerase. Mol Cell Biol.

[111] An S, Hu M (2022). Quercetin Promotes TFEB Nuclear Translocation and Activates Lysosomal Degradation of Ferritin to Induce Ferroptosis in Breast Cancer Cells. Comput Intell Neurosci.

[112] Yang Y, Lu Y, Zhang C, Guo Q, Zhang W, Wang T (2022). Phenazine derivatives attenuate the stemness of breast cancer cells through triggering ferroptosis. Cell Mol Life Sci.

[113] Lin YS, Shen YC, Wu CY, Tsai YY, Yang YH, Lin YY (2019). Danshen Improves Survival of Patients With Breast Cancer and Dihydroisotanshinone I Induces Ferroptosis and Apoptosis of Breast Cancer Cells. Front Pharmacol.

[114] Luo N, Zhang K, Li X, Hu Y, Guo L (2025). Tanshinone IIA destabilizes SLC7A11 by regulating PIAS4-mediated SUMOylation of SLC7A11 through KDM1A, and promotes ferroptosis in breast cancer. J Adv Res.

[115] Wu S, Wu X, Wang Q, Chen Z, Li L, Chen H (2024). Bufalin induces ferroptosis by modulating the 2,4-dienoyl-CoA reductase (DECR1)-SLC7A11 axis in breast cancer. Phytomedicine.

[116] Yin J, Lin Y, Fang W, Zhang X, Wei J, Hu G (2022). Tetrandrine citrate suppresses breast cancer via depletion of glutathione peroxidase 4 and activation of nuclear receptor coactivator 4-mediated ferritinophagy. Front Pharmacol.

[117] Zhai FG, Liang QC, Wu YY, Liu JQ, Liu JW (2022). Red ginseng polysaccharide exhibits anticancer activity through GPX4 downregulation-induced ferroptosis. Pharm Biol.

[118] DU X, Zhang J, Liu L, Xu B, Han H, Dai W (2022). A novel anticancer property of Lycium barbarum polysaccharide in triggering ferroptosis of breast cancer cells. J Zhejiang Univ Sci B.

[119] Chen HC, Tang HH, Hsu WH, Wu SY, Cheng WH, Wang BY (2022). Vulnerability of Triple-Negative Breast Cancer to Saponin Formosanin C-Induced Ferroptosis. Antioxidants (Basel).

[120] Huang G, Cai Y, Ren M, Zhang X, Fu Y, Cheng R (2025). Salidroside sensitizes Triple-negative breast cancer to ferroptosis by SCD1-mediated lipogenesis and NCOA4-mediated ferritinophagy. J Adv Res.

[121] Liu Y, Sun Q, Guo J, Yan L, Yan Y, Gong Y (2025). Dual ferroptosis induction in N2-TANs and TNBC cells via FTH1 targeting: A therapeutic strategy for triple-negative breast cancer. Cell Rep Med.

[122] Ye S, Hu X, Sun S, Su B, Cai J, Jiang J (2024). Oridonin promotes RSL3-induced ferroptosis in breast cancer cells by regulating the oxidative stress signaling pathway JNK/Nrf2/HO-1. Eur J Pharmacol.

[123] Yi N, Wang L, Jiang Z, Xu G, Li L, Zhang Y (2024). Peiminine triggers ferroptosis to inhibit breast cancer growth through triggering Nrf2 signaling. Tissue Cell.

[124] Yang X, Liang B, Zhang L, Zhang M, Ma M, Qing L (2024). Ursolic acid inhibits the proliferation of triple negative breast cancer stem like cells through NRF2 mediated ferroptosis. Oncol Rep.

[125] Yan C, Xuan F (2024). Paris saponin VII promotes ferroptosis to inhibit breast cancer via Nrf2/GPX4 axis. Biochem Biophys Res Commun.

[126] Niu X, Ding X, Tong Q, Huang X, Ma X, Li Z (2024). Myricetin inhibits 4 T1 breast tumor growth in mice via induction of Nrf-2/GPX4 pathway-mediated Ferroptosis. Toxicol Appl Pharmacol.

[127] Li Y, Lin H, Sun Y, Zhao R, Liu Y, Han J (2025). Platycodin D2 Mediates Incomplete Autophagy and Ferroptosis in Breast Cancer Cells by Regulating Mitochondrial ROS. Phytother Res.

[128] Sun C, Liu P, Pei L, Zhao M, Huang Y (2022). Propofol Inhibits Proliferation and Augments the Anti-Tumor Effect of Doxorubicin and Paclitaxel Partly Through Promoting Ferroptosis in Triple-Negative Breast Cancer Cells. Front Oncol.

[129] Li H, Liu W, Zhang X, Wu F, Sun D, Wang Z (2021). Ketamine suppresses proliferation and induces ferroptosis and apoptosis of breast cancer cells by targeting KAT5/GPX4 axis. Biochem Biophys Res Commun.

[130] Sun D, Li YC, Zhang XY (2021). Lidocaine Promoted Ferroptosis by Targeting miR-382-5p /SLC7A11 Axis in Ovarian and Breast Cancer. Front Pharmacol.

[131] Chen J, Qin C, Zhou Y, Chen Y, Mao M, Yang J (2022). Metformin may induce ferroptosis by inhibiting autophagy via lncRNA H19 in breast cancer. FEBS Open Bio.

[132] Yang J, Zhou Y, Xie S, Wang J, Li Z, Chen L (2021). Metformin induces Ferroptosis by inhibiting UFMylation of SLC7A11 in breast cancer. J Exp Clin Cancer Res.

[133] Hou Y, Cai S, Yu S, Lin H (2021). Metformin induces ferroptosis by targeting miR-324-3p/GPX4 axis in breast cancer. Acta Biochim Biophys Sin (Shanghai).

[134] Shen M, Cao S, Long X, Xiao L, Yang L, Zhang P (2024). DNAJC12 causes breast cancer chemotherapy resistance by repressing doxorubicin-induced ferroptosis and apoptosis via activation of AKT. Redox Biol.

[135] Yu X, Cheng L, Liu S, Wang M, Zhang H, Wang X (2024). Correlation between ferroptosis and adriamycin resistance in breast cancer regulated by transferrin receptor and its molecular mechanism. FASEB J.

[136] Li W, Guo Y, Xu Z, Li F, Dong Y, Xu F (2024). Notoginsenoside R1 (NGR1) regulates the AGE-RAGE signaling pathway by inhibiting RUNX2 expression to accelerate ferroptosis in breast cancer cells. Aging (Albany NY).

[137] Huang C, Guo Y, Li T, Sun G, Yang J, Wang Y (2024). Pharmacological activation of GPX4 ameliorates doxorubicin-induced cardiomyopathy. Redox Biol.

[138] Yao X, Xie R, Cao Y, Tang J, Men Y, Peng H (2021). Simvastatin induced ferroptosis for triple-negative breast cancer therapy. J Nanobiotechnology.

[139] Tang WJ, Xu D, Liang MX, Wo GQ, Chen WQ, Tang JH (2024). Pitavastatin induces autophagy-dependent ferroptosis in MDA-MB-231 cells via the mevalonate pathway. Heliyon.

[140] Mokhtarpour K, Razi S, Rezaei N (2024). Ferroptosis as a promising targeted therapy for triple negative breast cancer. Breast Cancer Res Treat.

[141] Lin Z, Liu Z, Yang X, Pan Z, Feng Y, Zhang Y (2024). Simeprevir induces ferroptosis through β-TrCP/Nrf2/GPX4 axis in triple-negative breast cancer cells. Biomed Pharmacother.

[142] Mathew M, Sivaprakasam S, Dharmalingam-Nandagopal G, Sennoune SR, Nguyen NT, Jaramillo-Martinez V (2024). Induction of oxidative stress and ferroptosis in triple-negative breast cancer cells by niclosamide via blockade of the function and expression of SLC38A5 and SLC7A11. Antioxidants (Basel).

[143] Gong G, Wan Y, Liu Y, Zhang Z, Zheng Y (2024). Ononin triggers ferroptosis-mediated disruption in the triple negative breast cancer both in vitro and in vivo. Int Immunopharmacol.

[144] Xiang L, Li Q, Guan Z, Wang G, Yu X, Zhang X, Zhang G (2024). Oxyresveratrol as a novel ferroptosis inducer exhibits anticancer activity against breast cancer via the EGFR/PI3K/AKT/GPX4 signalling axis. Front Pharmacol.

[145] Cao X, Li Y, Wang Y, Yu T, Zhu C, Zhang X (2022). Curcumin suppresses tumorigenesis by ferroptosis in breast cancer. PLoS One.

[146] Tang X, Ding H, Liang M, Chen X, Yan Y, Wan N (2021). Curcumin induces ferroptosis in non-small-cell lung cancer via activating autophagy. Thorac Cancer.

[147] Alkan AH, Ensoy M, Cansaran-Duman D (2024). A new therapeutic strategy for luminal A-breast cancer treatment: vulpinic acid as an anti-neoplastic agent induces ferroptosis and apoptosis mechanisms. Med Oncol.

[148] Beatty A, Singh T, Tyurina YY, Tyurin VA, Samovich S, Nicolas E (2021). Ferroptotic cell death triggered by conjugated linolenic acids is mediated by ACSL1. Nat Commun.

[149] Wen Y, Chen H, Zhang L, Wu M, Zhang F, Yang D, Shen J (2021). Glycyrrhetinic acid induces oxidative/nitrative stress and drives ferroptosis through activating NADPH oxidases and iNOS, and depriving glutathione in triple-negative breast cancer cells. Free Radic Biol Med.

[150] Lee H, Zandkarimi F, Zhang Y, Meena JK, Kim J, Zhuang L (2020). Energy-stress-mediated AMPK activation inhibits ferroptosis. Nat Cell Biol.

[151] Fang Q, Fang Q, Cheng R, Feng T, Xin W (2024). CAPE activates AMPK and Foxo3 signaling to induce growth inhibition and ferroptosis in triple-negative breast cancer. PLoS One.

[152] Zhou Y, Yang J, Chen C, Li Z, Chen Y, Zhang X (2021). Polyphyllin Ⅲ-Induced Ferroptosis in MDA-MB-231 Triple-Negative Breast Cancer Cells can Be Protected Against by KLF4-Mediated Upregulation of xCT. Front Pharmacol.

[153] Wei Y, Zhu Z, Hu H, Guan J, Yang B, Zhao H (2022). Eupaformosanin induces apoptosis and ferroptosis through ubiquitination of mutant p53 in triple-negative breast cancer. Eur J Pharmacol.

[154] Xie D, Jiang Y, Wang H, Zhu L, Huang S, Liu S (2024). Formononetin triggers ferroptosis in triple-negative breast cancer cells by regulating the mTORC1/SREBP1/SCD1 pathway. Front Pharmacol.

[155] Szeto GL, Finley SD (2019). Integrative Approaches to Cancer Immunotherapy. Trends Cancer.

[156] Keenan TE, Tolaney SM (2020). Role of Immunotherapy in Triple-Negative Breast Cancer. J Natl Compr Canc Netw.

[157] Emens L, Adams S, Barrios C, Dieras V, Iwata H, Loi S (2020). LBA16 IMpassion130: Final OS analysis from the pivotal phase III study of atezolizumab + nab-paclitaxel vs placebo + nab-paclitaxel in previously untreated locally advanced or metastatic triple-negative breast cancer. Annals of Oncology.

[158] Xie J, Yang A, Liu Q, Deng X, Lv G, Ou X (2024). Single-cell RNA sequencing elucidated the landscape of breast cancer brain metastases and identified ILF2 as a potential therapeutic target. Cell Prolif.

[159] Mezni E, Behi K, Gonçalves A (2022). Immunotherapy and breast cancer: an overview. Curr Opin Oncol.

[160] Zou Y, Zhang H, Liu F, Chen ZS, Tang H (2024). Intratumoral microbiota in orchestrating cancer immunotherapy response. J Transl Int Med.

[161] Majidpoor J, Mortezaee K (2021). The efficacy of PD- 1/PD-L1 blockade in cold cancers and future perspectives. Clin Immunol.

[162] Miles D, Gligorov J, André F, Cameron D, Schneeweiss A, Barrios C (2021). Primary results from IMpassion131, a double-blind, placebo-controlled, randomised phase III trial of first-line paclitaxel with or without atezolizumab for unresectable locally advanced/metastatic triple-negative breast cancer. Ann Oncol.

[163] Yang F, Xiao Y, Ding JH, Jin X, Ma D, Li DQ (2023). Ferroptosis heterogeneity in triple-negative breast cancer reveals an innovative immunotherapy combination strategy. Cell Metab.

[164] Wang W, Green M, Choi JE, Gijón M, Kennedy PD, Johnson JK (2019). CD8(+) T cells regulate tumour ferroptosis during cancer immunotherapy. Nature.

[165] Zhang C, Liu X, Jin S, Chen Y, Guo R (2022). Ferroptosis in cancer therapy: a novel approach to reversing drug resistance. Mol Cancer.

[166] Huang H, Wang G, Zeng D, Roche LAT, Zhuo R, Wilde RL (2025). Ultrasound genomics related mitochondrial gene signature for prognosis and neoadjuvant chemotherapy resistance in triple negative breast cancer. Oncol Res.

[167] Efimova I, Catanzaro E, Van der Meeren L, Turubanova VD, Hammad H, Mishchenko TA (2020). Vaccination with early ferroptotic cancer cells induces efficient antitumor immunity. J Immunother Cancer.

[168] Luo X, Gong HB, Gao HY, Wu YP, Sun WY, Li ZQ (2021). Oxygenated phosphatidylethanolamine navigates phagocytosis of ferroptotic cells by interacting with TLR2. Cell Death Differ.

[169] Kim DH, Kim WD, Kim SK, Moon DH, Lee SJ (2020). TGF-β1-mediated repression of SLC7A11 drives vulnerability to GPX4 inhibition in hepatocellular carcinoma cells. Cell Death Dis.

[170] Wang T, Zhang Y, Liu H, Wu J (2025). FOXM1 derived from triple-negative breast cancer exosomes promotes cancer progression by activating IDO1 transcription in macrophages to suppress ferroptosis and induce M2 polarization of tumor-associated macrophages. Genes Genet Syst.

[171] Tang B, Zhu J, Wang Y, Chen W, Fang S, Mao W (2023). Targeted xCT-mediated Ferroptosis and Protumoral Polarization of Macrophages Is Effective against HCC and Enhances the Efficacy of the Anti-PD-1/L1 Response. Adv Sci (Weinh).

[172] Zhou TJ, Zhang MM, Liu DM, Huang LL, Yu HQ, Wang Y (2024). Glutathione depletion and dihydroorotate dehydrogenase inhibition actuated ferroptosis-augment to surmount triple-negative breast cancer. Biomaterials.

[173] Wei R, Fu G, Li Z, Liu Y, Xue M (2024). Engineering Iron-Based Nanomaterials for Breast Cancer Therapy Associated with Ferroptosis. Nanomedicine (Lond).

[174] Zhang J, Zhang S, Liu M, Yang Z, Huang R (2024). Research Progress on Ferroptosis and Nanotechnology-Based Treatment in Triple-Negative Breast Cancer. Breast Cancer (Dove Med Press).

[175] Mao X, Tang X, Pan H, Yu M, Ji S, Qiu W (2024). B cells and IL-21-producing follicular helper t cells cooperate to determine the dynamic alterations of premetastatic tumor draining lymph nodes of breast cancer. Research (Wash D C).

[176] Luo L, Xu G (2022). Fascaplysin Induces Apoptosis and Ferroptosis, and Enhances Anti-PD-1 Immunotherapy in Non-Small Cell Lung Cancer (NSCLC) by Promoting PD-L1 Expression. Int J Mol Sci.

[177] Zhang Y, Shen G, Meng T, Lv Z, Li X, Li J (2023). Eicosapentaenoic acid enhances the sensitivity of osteosarcoma to cisplatin by inducing ferroptosis through the DNA-PKcs/AKT/NRF2 pathway and reducing PD-L1 expression to attenuate immune evasion. Int Immunopharmacol.

[178] Zhou J, Wan F, Wang L, Peng C, Huang R, Peng F (2020). STAT4 facilitates PD-L1 level via IL-12R/JAK2/STAT3 axis and predicts immunotherapy response in breast cancer. MedComm.

[179] Desterke C, Xiang Y, Elhage R, Duruel C, Chang Y, Hamaï A (2023). Ferroptosis Inducers Upregulate PD-L1 in Recurrent Triple-Negative Breast Cancer. Cancers (Basel).

[180] Cha JH, Chan LC, Li CW, Hsu JL, Hung MC (2019). Mechanisms Controlling PD-L1 Expression in Cancer. Mol Cell.

[181] Yamaguchi H, Hsu JM, Yang WH, Hung MC (2022). Mechanisms regulating PD-L1 expression in cancers and associated opportunities for novel small-molecule therapeutics. Nat Rev Clin Oncol.

[182] Dodson M, Castro-Portuguez R, Zhang DD (2019). NRF2 plays a critical role in mitigating lipid peroxidation and ferroptosis. Redox Biol.

[183] Ouyang S, Li H, Lou L, Huang Q, Zhang Z, Mo J (2022). Inhibition of STAT3-ferroptosis negative regulatory axis suppresses tumor growth and alleviates chemoresistance in gastric cancer. Redox Biol.

[184] Zhang W, Gong M, Zhang W, Mo J, Zhang S, Zhu Z (2022). Correction: Thiostrepton induces ferroptosis in pancreatic cancer cells through STAT3/GPX4 signalling. Cell Death Dis.

[185] Dai Y, Cui C, Jiao D, Zhu X (2025). JAK/STAT signaling as a key regulator of ferroptosis: mechanisms and therapeutic potentials in cancer and diseases. Cancer Cell Int.

[186] Lang X, Green MD, Wang W, Yu J, Choi JE, Jiang L (2019). Radiotherapy and immunotherapy promote tumoral lipid oxidation and ferroptosis via synergistic repression of SLC7A11. Cancer Discov.

[187] Liao P, Wang W, Wang W, Kryczek I, Li X, Bian Y (2022). CD8(+) T cells and fatty acids orchestrate tumor ferroptosis and immunity via ACSL4. Cancer Cell.

[188] Kobayashi KS, van den Elsen PJ (2012). NLRC5: a key regulator of MHC class I-dependent immune responses. Nat Rev Immunol.

[189] Lai Y, Gao Y, Lin J, Liu F, Yang L, Zhou J (2024). Dietary elaidic acid boosts tumoral antigen presentation and cancer immunity via ACSL5. Cell Metab.

[190] Lei G, Horbath A, Li Z, Gan B (2022). PKCβII-ACSL4 pathway mediating ferroptosis execution and anti-tumor immunity. Cancer Commun (Lond).

[191] Zhang HL, Hu BX, Li ZL, Du T, Shan JL, Ye ZP (2022). PKCβII phosphorylates ACSL4 to amplify lipid peroxidation to induce ferroptosis. Nat Cell Biol.

[192] Liu S, Fan S, Wang Y, Chen R, Wang Z, Zhang Y (2023). ACSL4 serves as a novel prognostic biomarker correlated with immune infiltration in Cholangiocarcinoma. BMC Cancer.

[193] Anestis A, Zoi I, Papavassiliou AG, Karamouzis MV (2020). Androgen Receptor in Breast Cancer-Clinical and Preclinical Research Insights. Molecules.

[194] Liang D, Feng Y, Zandkarimi F, Wang H, Zhang Z, Kim J (2023). Ferroptosis surveillance independent of GPX4 and differentially regulated by sex hormones. Cell.

[195] Sun H, Zhang L, Wang Z, Gu D, Zhu M, Cai Y (2023). Single-cell transcriptome analysis indicates fatty acid metabolism-mediated metastasis and immunosuppression in male breast cancer. Nat Commun.

[196] Ravaioli S, Maltoni R, Pasculli B, Parrella P, Giudetti AM, Vergara D (2022). Androgen receptor in breast cancer: The “5W” questions. Front Endocrinol (Lausanne).

[197] Kolyvas EA, Caldas C, Kelly K, Ahmad SS (2022). Androgen receptor function and targeted therapeutics across breast cancer subtypes. Breast Cancer Res.

[198] Garcia-Martinez L, Zhang Y, Nakata Y, Chan HL, Morey L (2021). Epigenetic mechanisms in breast cancer therapy and resistance. Nat Commun.

[199] Hogg SJ, Beavis PA, Dawson MA, Johnstone RW (2020). Targeting the epigenetic regulation of antitumour immunity. Nat Rev Drug Discov.

[200] Jones PA, Ohtani H, Chakravarthy A, De Carvalho DD (2019). Epigenetic therapy in immune-oncology. Nat Rev Cancer.

[201] Devaiah BN, Case-Borden C, Gegonne A, Hsu CH, Chen Q, Meerzaman D (2016). BRD4 is a histone acetyltransferase that evicts nucleosomes from chromatin. Nat Struct Mol Biol.

[202] Liang D, Yu Y, Ma Z (2020). Novel strategies targeting bromodomain-containing protein 4 (BRD4) for cancer drug discovery. Eur J Med Chem.

[203] Ding R, Tang L, Zeng D, Li J, Jia Y, Yan X (2025). Discovery of novel JQ1 derivatives as dual ferroptosis and apoptosis inducers for the treatment of triple-negative breast cancer. Eur J Med Chem.

[204] Zhang Y, Zheng L, Ma L, Yin F, Luo Z, Li S (2024). Discovery of dual CDK6/ BRD4 inhibitor inducing apoptosis and increasing the sensitivity of ferroptosis in triple-negative breast cancer. J Med Chem.

[205] Zhu X, Fu Z, Dutchak K, Arabzadeh A, Milette S, Steinberger J (2024). Cotargeting CDK4/6 and BRD4 Promotes Senescence and Ferroptosis Sensitivity in Cancer. Cancer Res.

[206] Esquea EM, Young RG, Ciraku L, Merzy J, Ahmed NN, Talarico AN (2024). ACSS2 regulates ferroptosis in an E2F1-dependent manner in breast cancer brain metastatic cells. bioRxiv.

[207] Davies A, Nouruzi S, Ganguli D, Namekawa T, Thaper D, Linder S (2021). An androgen receptor switch underlies lineage infidelity in treatment-resistant prostate cancer. Nat Cell Biol.

[208] Kim J, Lee Y, Lu X, Song B, Fong KW, Cao Q, Licht JD, Zhao JC, Yu J (2018). Polycomb- and Methylation-Independent Roles of EZH2 as a Transcription Activator. Cell Rep.

[209] Xiao G, Jin LL, Liu CQ, Wang YC, Meng YM, Zhou ZG (2019). EZH2 negatively regulates PD-L1 expression in hepatocellular carcinoma. J Immunother Cancer.

[210] Huang J, Yin Q, Wang Y, Zhou X, Guo Y, Tang Y (2024). EZH2 inhibition enhances PD-L1 protein stability through USP 22-mediated deubiquitination in colorectal cancer. Adv Sci (Weinh).

[211] Zhao Y, Wang XX, Wu W, Long H, Huang J, Wang Z (2019). EZH2 regulates PD-L1 expression via HIF-1α in non-small cell lung cancer cells. Biochem Biophys Res Commun.

[212] Lai Y, Han X, Xie B, Xu Y, Yang Z, Wang D (2024). EZH2 suppresses ferroptosis in hepatocellular carcinoma and reduces sorafenib sensitivity through epigenetic regulation of TFR2. Cancer Sci.

[213] Lee J, You C, Kwon G, Noh J, Lee K, Kim K (2024). Integration of epigenomic and transcriptomic profiling uncovers EZH2 target genes linked to cysteine metabolism in hepatocellular carcinoma. Cell Death Dis.

[214] Pan G, Xia Y, Hao M, Guan J, Zhu Q, Zha T (2025). EZH2 suppresses IR-induced ferroptosis by forming a co-repressor complex with HIF-1α to inhibit ACSL4: Targeting EZH2 enhances radiosensitivity in KDM6A-deficient esophageal squamous carcinoma. Cell Death Differ.

[215] Hwang JW, Cho Y, Bae GU, Kim SN, Kim YK (2021). Protein arginine methyltransferases: promising targets for cancer therapy. Exp Mol Med.

[216] Jarrold J, Davies CC (2019). PRMTs and Arginine Methylation: Cancer’s Best-Kept Secret?. Trends Mol Med.

[217] Feustel K, Falchook GS (2022). Protein Arginine Methyltransferase 5 (PRMT5) Inhibitors in Oncology Clinical Trials: A review. J Immunother Precis Oncol.

[218] Fan Y, Wang Y, Dan W, Zhang Y, Nie L, Ma Z (2025). PRMT5-mediated arginine methylation stabilizes GPX4 to suppress ferroptosis in cancer. Nat Cell Biol.

[219] Zhang X, Duan Y, Li S, Zhang Z, Peng L, Ma X (2024). CRISPR screening identifies PRMT1 as a key pro-ferroptotic gene via a two-layer regulatory mechanism. Cell Rep.

[220] Deng N, Qiao M, Li Y, Liang F, Li J, Liu Y (2023). Anticancer effects of licochal-cones: A review of the mechanisms. Front Pharmacol.

[221] Zhang JX, Xiao Y, Li YQ, Zhu YL, Li YR, Zhao RS (2023). Licochalcone A induces ferroptosis in hepatocellular carcinoma via reactive oxygen species activated by the SLC7A11/GPX4 pathway. Integr Cancer Ther.

[222] Gong S, Maegawa S, Yang Y, Gopalakrishnan V, Zheng G, Cheng D (2020). Licochalcone A is a Natural Selective Inhibitor of Arginine Methyltransferase 6. Biochem J.

[223] Zeng W, Zhang R, Huang P, Chen M, Chen H, Zeng X (2025). Ferroptotic Neutrophils Induce Immunosuppression and Chemoresistance in Breast Cancer. Cancer Res.

[224] Rossi Sebastiano M, Pozzato C, Saliakoura M, Yang Z, Peng RW, Galiè M (2020). ACSL3-PAI-1 signaling axis mediates tumor-stroma cross-talk promoting pancreatic cancer progression. Sci Adv.

[225] Wang Y, An J, Zhou J, Chang L, Zhang Q, Peng F (2024). Hydroxysafflor yellow A: a natural pigment with potential anticancer therapeutic effect. Front Pharmacol.

[226] Catanzaro E, Demuynck R, Naessens F, Galluzzi L, Krysko DV (2024). Immunogenicity of ferroptosis in cancer: a matter of context?. Trends Cancer.

[227] Xu Y, Ge M, Xu Y, Yin K (2025). Ferroptosis: a novel perspective on tumor immunotherapy. Front Immunol.

[228] Xiang S, Yan W, Ren X, Feng J, Zu X (2024). Role of ferroptosis and ferroptosis-related long non’coding RNA in breast cancer. Cell Mol Biol Lett.

[229] Hushmandi K, Klionsky DJ, Aref AR, Bonyadi M, Reiter RJ, Nabavi N (2024). Ferroptosis contributes to the progression of female-specific neoplasms, from breast cancer to gynecological malignancies in a manner regulated by non-coding RNAs: Mechanistic implications. Noncoding RNA Res.

[230] Shang Y, Zhao M, Chen S, Chen Y, Liu X, Zhou F (2024). Tetrastigma hemsleyanum polysaccharide combined with doxorubicin promote ferroptosis and immune function in triple-negative breast cancer. Int J Biol Macromol.

[231] Zhang S, Guo L, Tao R, Liu S (2025). Ferroptosis-targeting drugs in breast cancer. J Drug Target.

[232] Peng C, Chen Y, Jiang M (2024). Targeting ferroptosis: a promising strategy to overcome drug resistance in breast cancer. Front Oncol.

[233] Yang Y, Yu S, Liu W, Zhuo Y, Qu C, Zeng Y (2025). Ferroptosis-related signaling pathways in cancer drug resistance. Cancer Drug Resist.

[234] Yuan W, Guifang Y, Xin C (2024). Mechanism of ferroptosis resistance in cancer cells. Cancer Drug Resistance.

[235] Ge A, Xiang W, Li Y, Zhao D, Chen J, Daga P (2024). Broadening horizons: the multifaceted role of ferroptosis in breast cancer. Front Immunol.

[236] Ge A, He Q, Zhao D, Li Y, Chen J, Deng Y (2024). Mechanism of ferroptosis in breast cancer and research progress of natural compounds regulating ferroptosis. J Cell Mol Med.

[237] Hu S, Chu Y, Zhou X, Wang X (2023). Recent advances of ferroptosis in tumor: From biological function to clinical application. Biomed Pharmacother.

[238] Sun S, Shen J, Jiang J, Wang F, Min J (2023). Targeting ferroptosis opens new avenues for the development of novel therapeutics. Signal Transduct Target Ther.

[239] Gong D, Chen M, Wang Y, Shi J, Hou Y (2022). Role of ferroptosis on tumor progression and immunotherapy. Cell Death Discov.

